# Proteomic identification and characterization of hepatic glyoxalase 1 dysregulation in non-alcoholic fatty liver disease

**DOI:** 10.1186/s12953-018-0131-y

**Published:** 2018-02-14

**Authors:** Christos Spanos, Elaina M. Maldonado, Ciarán P. Fisher, Petchpailin Leenutaphong, Ernesto Oviedo-Orta, David Windridge, Francisco J. Salguero, Alexandra Bermúdez-Fajardo, Mark E. Weeks, Caroline Evans, Bernard M. Corfe, Naila Rabbani, Paul J. Thornalley, Michael H. Miller, Huan Wang, John F. Dillon, Alberto Quaglia, Anil Dhawan, Emer Fitzpatrick, J. Bernadette Moore

**Affiliations:** 1 Department of Nutritional Sciences, Faculty of Health and Medical Sciences, University of Surrey, Guildford, Surrey, GU2 7XH UK; 2 Institute of Child Health, University College London, WC1N 1EH, London, UK; 3 Biological and Systems Engineering Group, ChELSI Institute, Department of Chemical and Biological Engineering, University of Sheffield, S1 3JD, Sheffield, UK; 4 Molecular Gastroenterology Research Group, Department of Oncology and Insigneo Institute for in silico Medicine, University of Sheffield, S10 2RX, Sheffield, UK; 5 Clinical Sciences Research Laboratories, Warwick Medical School, University of Warwick, University Hospital, Coventry, CV2 2DX UK; 6 Medical Research Institute, University of Dundee, Ninewells Hospital and Medical School, Dundee, DD1 9SY UK; 7 Paediatric Liver, GI and Nutrition Centre, King’s College London School of Medicine, London, SE5 9RS UK; 8 School of Food Science and Nutrition, University of Leeds, Leeds, LS2 9JT UK

**Keywords:** Non-alcoholic fatty liver disease, Glyoxalase, Methylglyoxal, Proteomics, iTRAQ

## Abstract

**Background:**

Non-alcoholic fatty liver disease (NAFLD) is the most common liver disease worldwide. However, its molecular pathogenesis is incompletely characterized and clinical biomarkers remain scarce. The aims of these experiments were to identify and characterize liver protein alterations in an animal model of early, diet-related, liver injury and to assess novel candidate biomarkers in NAFLD patients.

**Methods:**

Liver membrane and cytosolic protein fractions from high fat fed apolipoprotein E knockout (ApoE^−/−^) animals were analyzed by quantitative proteomics, utilizing isobaric tags for relative and absolute quantitation (iTRAQ) combined with nano-liquid chromatography and tandem mass spectrometry (nLC-MS/MS). Differential protein expression was confirmed independently by immunoblotting and immunohistochemistry in both murine tissue and biopsies from paediatric NAFLD patients. Candidate biomarkers were analyzed by enzyme-linked immunosorbent assay in serum from adult NAFLD patients.

**Results:**

Through proteomic profiling, we identified decreased expression of hepatic glyoxalase 1 (GLO1) in a murine model. GLO1 protein expression was also found altered in tissue biopsies from paediatric NAFLD patients. In vitro experiments demonstrated that, in response to lipid loading in hepatocytes, GLO1 is first hyperacetylated then ubiquitinated and degraded, leading to an increase in reactive methylglyoxal. In a cohort of 59 biopsy-confirmed adult NAFLD patients, increased serum levels of the primary methylglyoxal-derived advanced glycation endproduct, hydroimidazolone (MG-H1) were significantly correlated with body mass index (*r* = 0.520, *p* < 0.0001).

**Conclusion:**

Collectively these results demonstrate the dysregulation of GLO1 in NAFLD and implicate the acetylation-ubquitination degradation pathway as the functional mechanism. Further investigation of the role of GLO1 in the molecular pathogenesis of NAFLD is warranted.

**Electronic supplementary material:**

The online version of this article (10.1186/s12953-018-0131-y) contains supplementary material, which is available to authorized users.

## Background

Non-alcoholic fatty liver disease (NAFLD) is increasingly the most common chronic liver disease in developed nations, with an estimated global adult prevalence of 25% [1]. Closely associated with obesity, NAFLD is also an escalating problem both in developing countries [2] and in children [3, 4]. The term NAFLD encompasses a range of histopathologies from simple steatosis to non-alcoholic steatohepatitis (NASH). Whereas steatosis is considered relatively benign and potentially reversible, NASH can progress to irreversible fibrosis and cirrhosis, leading to an increased risk of mortality from liver-related and cardiovascular-related causes [5]. A natural history study of NAFLD in children over 20 years demonstrated that children with NASH can also progress to advanced fibrosis, cirrhosis and ultimately end-stage liver disease and death [6]. Due to its increasing burden on health services worldwide, early screening and diagnosis of NAFLD is urgently required.

The pathogenesis of NAFLD is clearly multifactorial with both genetic and environmental factors influencing the development and progression of the disease [7]. Current estimates suggest the heritability of NAFLD is around 39% and several genetic polymorphisms associated with disease risk have been identified [8, 9]. Risk factors for NAFLD include obesity, insulin resistance and hyperlipidemia, with a clear role for diet and physical activity as key modifiable risk factors [10, 11]. Despite substantial research, the multifactorial nature of NAFLD has prevented complete characterization of this disease and the molecular mechanisms that mediate its pathogenesis. This has contributed to the shortage of effective molecular biomarkers for early diagnosis and accurate disease staging of NAFLD.

Although tools such as liver function tests, imaging and complex scoring systems can be useful in decision algorithms for triaging patients for liver biopsy, there are currently no non-invasive tests that can completely replace invasive liver biopsy for the detection of NASH [12]. Ongoing developments in magnetic resonance-based imaging techniques are promising, but still require standardization and validation, and are unlikely to be widely available in the near future due to cost [13, 14]. In parallel, mass spectrometry-based proteomic approaches have evolved considerably in the last decade and offer the power to both identify disease biomarkers and drug targets while yielding insight into molecular mechanisms of a clinical condition [15, 16]. In the context of NAFLD, it is anticipated that a panel of serum biomarkers, combined with imaging techniques such as transient elastography, will ultimately provide optimal diagnostic discrimination of NAFLD, and facilitate the development of NAFLD-specific pharmacotherapies [17].

Glyoxalase 1 (GLO1) has been implicated in obesity-related conditions [18]. It is a critical cytosolic enzyme involved in the detoxification of reactive dicarbonyls, principally methylglyoxal (MG). MG is a major source of advanced glycation end products (AGE) including the highly reactive MG-derived hydroimidazolones (MG-Hs), these in turn induce sustained activation of AGE immune receptors (RAGE) in the circulation. Disruption of the glyoxalse system and a buildup of MG and MG-Hs is thought to be central in atherosclerosis and the long-term vascular complications of diabetes [19]. Animal models suggest GLO1 activity in the liver is decreased in response to both high fructose and high fat diets [20] [21], and increased AGEs in liver have been associated with steatosis severity in NAFLD patients [22]. Despite these associations, the specific role of GLO1 in the pathogenesis of NAFLD is unknown.

In this study, we employed quantitative proteomic profiling, utilizing advanced mass spectrometry techniques to evaluate altered expression patterns of hepatic proteins in a murine model of atherosclerosis and early liver injury. By adopting this hypothesis-generating approach, we identified GLO1 as a candidate biomarker for NAFLD. The results were validated through a comprehensive range of in vitro and in vivo experiments involving animal models, HepG2 cells and human hepatic biopsy samples. The underlying molecular mechanisms and functional pathways were explored in vitro*,* and the association of MG-H1 and sRAGE with liver injury was examined in serum collected from a cohort of adult patients with histologically confirmed NAFLD.

## Results

### Proteome analysis identifies glyoxalase 1 as a candidate biomarker for NAFLD

Isobaric tags for relative and absolute quantitation (iTRAQ) combined with nano-liquid chromatography and tandem mass spectrometry (nLC-MS/MS) was used to compare expression profiles of cytosolic and membrane proteins extracted from the livers of apolipoprotein E knockout (ApoE^−/−^) and wild type (WT) mice fed either a normal chow diet (ND) or a high fat diet (HFD) for a 12-week period (*n* = 3/group). The choice of a 12-week time point in this animal model had been judged as described below. The experimental workflow is illustrated in Fig. S1 (Additional file 1: Figure S1).

**Fig. 1:**
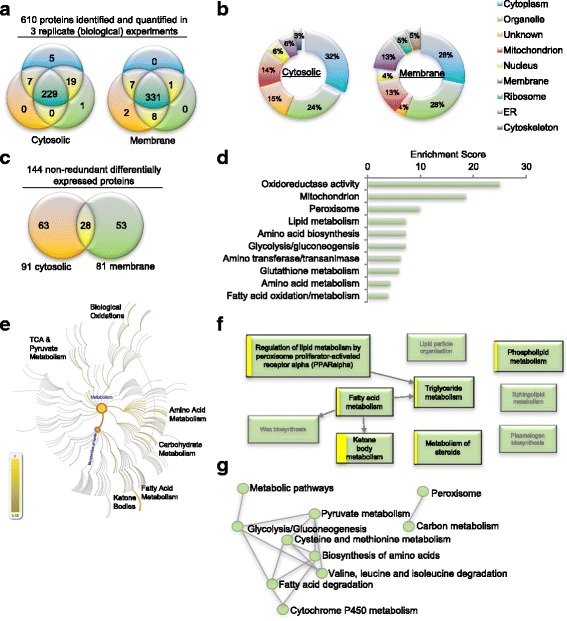
iTRAQ-based proteomic analysis of murine liver membrane and cytosolic proteins. **a** Venn analysis of proteins stringently identified and quantified by Scaffold_4.8.4 Q+, across biological replicates. Minimum thresholds for protein identification were set at 99% probability and at least 2 unique identified peptides. Protein probabilities were assigned by the Protein Prophet algorithm [57] and the calculated FDR for protein identification was < 0.05%. **b** Annotation of Gene Ontology (GO) category ‘cellular component ‘. **c** 91 cytosolic and 82 membranous proteins were identified significantly (*P* < 0.05) differentially expressed in all biological replicates, based on both randomized permutation and Kruskal-Wallis tests with Benjamini-Hochberg FDR for multiple testing corrections. 28 proteins were found in extracts from both cellular compartments. **d** Top functional annotation clusters, derived from multiple public sources of protein and gene annotation (eg GO, Uniprot, KEGG) utilizing the DAVID [23] knowledgebase, identified significantly enriched among the differentially expressed proteins. **e** Pathway topological analysis of the Reactome knowledgebase [25] illustrating pathway overrepresentation as corrected probability; intensity of scale indicates FDR. The lipid metabolism node is highlighted (in orange with blue writing) branching from overall metabolism node. **f** Dysregulated pathways branching from the lipid metabolism node. Box size is proportional to the entities in the Reactome pathway; the yellow band is proportional to the number of differentially expressed proteins that match against the query dataset; pathways over-represented are bolded with black text, shaded boxes represent pathways not found enriched. **g** Connectivity of enriched KEGG knowledgebase [27] pathways identified by Enrichr [26] analysis.

Proteomic analysis returned > 8000 peptides with a labelling efficiency ≥98% for each group (Additional file 1: Figure S2). Utilizing a protein identification threshold of at minimum 99% probability and ≥2 unique peptides, Scaffold calculated a Prophet false discovery rate (FDR) of < 0.05%. A total of 610 proteins were identified and quantified from 4447 uniquely assigned peptides (Additional files 2 and 3). Subsequent analyses focused on the 560 (229 cytosolic and 331 membrane) proteins found in all biological replicates (Fig. 1a). Gene Ontology (GO) analyses confirmed that cytoskeletal proteins were detected in the cytosolic fractions and membrane, ribosomal and endoplasmic reticulum (ER) proteins were detected in the membrane fraction (Fig. 1b), thereby verifying the enrichment strategy. 91 cytosolic and 81 membranous proteins were identified as significantly differentially expressed (*P* < 0.05) based on both randomized permutation and Kruskal-Wallis tests with Benjamini-Hochberg FDR used for multiple testing corrections (Additional file 1: Tables S1 and S2). Of 144 unique proteins, 28 proteins were identified in both cytosolic and membrane fractions (Fig. 1c). Although these fractions included proteins known to exist in multiple cellular locations, such as liver fatty acid binding protein (L-FABP), this result was also attributed to the use of differential centrifugation as an enrichment strategy, as opposed to a method of absolute fractionation.

Bioinformatic analyses were done using a variety of open source tools against several well curated databases. We used the Database for Annotation, Visualization and Integrated Discovery (DAVID) [23] for converting protein accession identifiers to gene symbols and for functional annotation clustering. DAVID released a completely rebuilt knowledgebase (version 6.8) in October 2016 overcoming previous criticism of being outdated [24]. For functional annotation clustering, DAVID’s algorithim clusters groups across multiple databases with differing biological focuses and annotation; eg. the Universal Protein Resource (UniProt), the Kyoto Encyclopedia of Genes and Genomes (KEGG) and the GO databases). The DAVID enrichment score is the geometric mean of modified Fisher Exact tests, where multiple testing correction issues are considered, associated with the enriched annotation terms that belong to the group. This analysis identified 33 functional clusters significantly associated with the proteins identified as dysregulated in this study. Oxidoreductase activity, mitochondria and peroxisome, terms indicative of an increased flux in fatty acid metabolism, were found most significantly enriched (Fig. 1d). Disruption of lipid, carbohydrates and amino acid metabolism was also highlighted within the ten most significant functional clusters (Fig. 1d).

Independent analysis of the Reactome pathway knowledgebase [25] identified metabolism as the most overrepresented pathway in the dysregulated proteins. Pathway topological analysis illustrates the corrected probability for pathway overrepresentation (Fig. 1e; intensity of scale indicates the Reactome pathway FDR). Significantly overrepresented pathways found associated with the differentially expressed proteins included: lipid, fatty acid, carbohydrate, tricarboxylic acid cycle (TCA), pyruvate and amino acid metabolic pathways; along with ketone bodies and biological oxidation pathways (Fig. 1e). Inspection of pathways branching from the lipid metabolism node highlighted in Fig. 1e, shows that peroxisome proliferator-activated receptor (PPAR) regulated pathways in triglyceride, fatty acid and ketone metabolism were significantly overrepresented (bolded boxes, Fig. 1f). In this representation, box size is proportional to the entities associated with the Reactome pathway, and the yellow band is proportional to the number of differentially expressed proteins that match against the query dataset (Fig. 1f). Specificity is observed here, as some pathways within lipid metabolism, namely lipid particle organization, sphingolipid metabolism and plasmalogen and wax biosynthesis were not found enriched (shaded boxes, Fig. 1f). Lastly, we used the recently updated enrichment analysis tool Enrichr [26] to examine the KEGG database [27]. In common with both the DAVID and Reactome analyses, fatty acid, amino acid, carbohydrate metabolic pathways were found significantly overrepresented, and connections between the top ten most significantly overrepresented pathways were visualized in a network diagram (Fig. 1g). PPAR signalling was also identified in the Enrichr analysis as overrepresented, and ranked 11th in the 20 most significantly overrepresented pathways identified. The differentially expressed proteins associated with the top 20 pathways were clustered both by enriched terms and input protein using the combined score, and visualized as a clustergram (Additional file 1: Figure S3). Collectively, these bioinformatic analyses infer widespread disturbances in intermediary metabolism in particular lipid, carbohydrate and amino acid metabolism.

Among others, L-FABP and fatty acid synthase (FASN) were also found to be significantly downregulated in the cytolosic fraction from the livers of ApoE^−/−^ HFD mice relative to their respective controls (− 0.9, *P* < 0.0001; and − 1.15, *P* < 0.0001, respectively; Additional file 1: Table S1). Both these proteins have been established as disrupted in patients with NAFLD [28, 29], suggesting some sensitivity of our approach to identify candidate tissue biomarkers relevant to liver injury in human NAFLD. Of note, the enzyme lactoylglutathione lyase (E.C.4.4.1.5), commonly known as GLO1, was also identified as differentially expressed (Additional file 1: Figure S4). This was of interest as we had independently identified GLO1 in pilot proteomic experiments as downregulated in fatty acid treated HuH7 cells [30]. Given the association between GLO1 and obesity and diabetes-related conditions, it represented an intriguing candidate for further investigation.

### ApoE^−/−^ mice develop liver injury in response to a high cholesterol and high fat diet

In a previous study, Tous and coworkers first demonstrated that ApoE^−/−^ mice develop hepatic steatosis in response to 10 weeks on a high-fat (20% palm oil) and high cholesterol (0.1% *w*/w cholesterol) diet [31]. Then in examining pathology in a time course after 5, 10, and 16 weeks on a high palm oil diet, the authors report a peak in both hepatic steatosis and atheroma burden after 10 weeks on diet, with pathology in 16-week-fed animals appearing to resolve back to levels observed in 5-week-fed mice [31]. However, there were no comparisons drawn to animals fed standard chow at each of the time points. In light of these results we designed our 16-week feeding protocol to have 4 equally spaced time points (after 4, 8, 12 and 16 weeks of diet) with control groups at each time point, and aimed to explore a time-point that represented early liver injury.

**Fig. 2:**
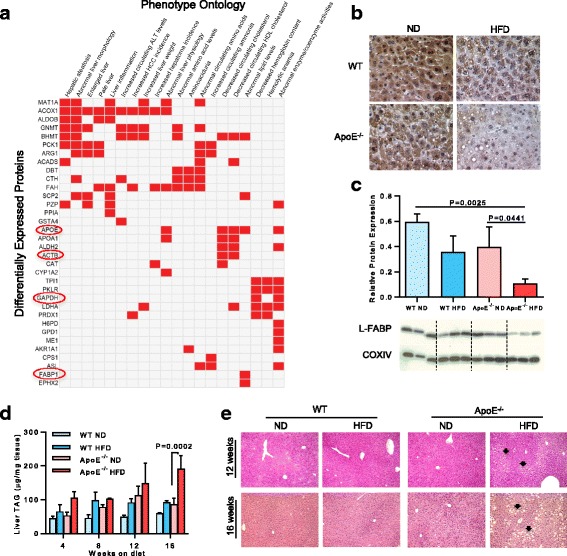
Hepatic pathology in high fat fed ApoE^−/−^ mice. **a** Clustergram of gene names for the differentially expressed proteins found significantly overrepresented in associated MGI Mammalian Phenotype Ontology [61]. Data are shown for top 20 enriched phenotypes, with both phenotypes and differentially expressed proteins clustered according to the combined statistical score. **b** Immunohistochemical assay of L-FABP expression in animals fed diets for 12 weeks. **c** Immunoblotting and quantification of L-FABP expression normalized to COXIV. Data are mean ± SD; *n* = 3 animals/group. **d** Liver lipids were quantified by triacylglycerol assay. **e** Histology shows that liver sections from ApoE^−/−^ HFD mice exhibit minimal to mild steatosis (arrows) at 12 and 16 weeks. Sections were stained with hematoxylin-eosin; original magnification: 100×

In examining the proteomic profiles of animals fed ND or HFD for 12 weeks against the Mouse Genome Informatics (MGI) Mammalian Phenotype Ontology, the top 20 most significantly enriched phenotypes were all indicative of liver injury and/or altered circulating lipid metabolites (Fig. 2a). The five most significant enriched ontologies were: ‘hepatic steatosis’, ‘abnormal liver morphology’, ‘enlarged liver’, ‘pale liver’, and ‘liver inflammation’ (Fig. 2a). Several enzymes involved in methylation reactions clustered highly significantly with liver injury, these included: methionine adenosyltransferase, glycine N-methyltransferas and betaine--homocysteine S-methyltransferase (MAT1a, GNMT and BHMT in Fig. 2a). In addition, key enzymes associated with fatty acid beta-oxidation, glycolysis and gluconeogenesis: acyl-CoA oxidase, aldolase B, and phosphoenolpyruvate carboxykinase 1 (ACOX1, ALDOB, PCK1 in Fig. 2a) were dysregulated and associated with liver injury. The iTRAQ experiment detected APOE as ‘downregulated’ in the liver membrane fraction from ApoE^−/−^ animals (− 1.15 and − 1.89, *P* < 0.0051; Fig. 2a; Additional file 1: Table S2). This serves to illustrate some of the well-known issues with iTRAQ underestimation and its limitations for precise, as opposed to relative, quantitation [32].

Interestingly, beta actin, glyceraldehyde 3-phosphate dehydrogenase and liver fatty acid binding protein (ACTB, GAPDH and FABP1 in Fig. 2a) were all identified as dysregulated in our dietary study. We independently observed a decrease in the detection of L-FABP protein in the livers of ApoE^−/−^ HFD mice fed for 12 weeks by immunohistochemistry assay (Fig. 2b). When measured quantitatively by immunoblotting, L-FABP levels in ApoE^−/−^ HFD mice were fourfold lower relative to those in ApoE^−/−^ ND mice (*P* = 0.0441), and sixfold lower relative to those in WT ND mice (*P* = 0.0025; Fig. 2C). Because ACTB and GAPDH, often used as ‘housekeeping’ internal controls, protein expression was altered by HFD and cytochrome c oxidase subunit 4 (COXIV) was identified as unregulated (*P* = 0.92, Additional file 3), we utilized COXIV for the immunoblotting internal control in downstream experiments. In sum, these bioinformatic analyses point to liver injury caused by the disruption of intermediary metabolism, in particular pathways involved in lipid, carbohydrate and amino acid metabolism. This is notably accompanied by dysregulation of proteins commonly thought of as ‘housekeeping’.

While levels of liver triacylglycerol (TAG) were higher in the ApoE^−/−^ HFD mice at all time points measured, by 16-weeks these were twofold higher than those in ApoE^−/−^ ND mice (*P* = 0.0002, Fig. 2d). In general, the histological phenotype in the liver was mild. Visualization of hematoxylin and eosin (H&E) and Masson’s trichrome stained liver sections showed that ApoE^−/−^ HFD mice developed only very mild steatosis without visible fibrosis after 12 weeks (Fig. 2e; and Additional file 1: Figure S5a, respectively). After 16 weeks, while more pronounced macrovesicular steatosis was evident, there was no histological evidence of fibrosis (Fig. 2e; Additional file 1: Figure S5a). In contrast, the ApoE^−/−^ HFD mice developed significant aortic sinus plaques after 12 weeks, with a plaque area that was more than threefold higher compared to that found in ApoE^−/−^ ND mice (Additional file 1: Figures S5b & S5c). In comparison, no visible plaques were detected in WT mice fed either ND or HFD at any of the time points, and at 16 weeks, the plaque area was >fourfold higher than that in ApoE^−/−^ ND mice (Additional file 1: Figures S5b & S5c).

While the liver histology was mild, nonetheless the changes in liver TAG levels and L-FABP expression, along with our proteomic data, suggested that the 12-week feeding regime was a valid time point for proteomic analyses targeted at identifying perturbations in pathways involved in the early stages of liver injury including NAFLD pathogenesis.

### Expression of glyoxalase 1 was altered in an animal model of early liver injury

**Fig. 3:**
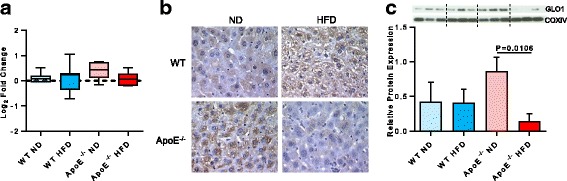
Altered GLO1 expression in animal model of early liver injury. **a** Median and interquartile range of 10 peptides for GLO1, quantified by iTRAQ-based proteomics. Whiskers are Tukey’s. **b** Immunohistochemical assay of GLO1 expression in WT and ApoE−/− animals fed a normal (ND) or high fat diet (HFD) for 12 weeks. **c** Immunoblotting and quantification showing GLO1 protein expression relative to COXIV. Data are mean ± SD, *n* = 3 animals/group

Differential expression of GLO1 in ApoE^−/−^ mice fed a high fat diet, as detected by iTRAQ (Fig. 3a), was independently verified by immunohistochemistry. The levels of positive GLO1 staining in liver sections taken from ApoE^−/−^ ND mice and WT HFD were higher than those from WT ND mice; whereas, the staining levels of GLO1 in liver sections from ApoE^−/−^ HFD mice were considered negligible (Fig. 3b). Densitometric analysis of immunoblots showed a twofold increase in the expression of GLO1 protein between ApoE^−/−^ ND and WT mice, and a fourfold decrease between that in ApoE^−/−^ HFD and ApoE^−/−^ ND mice (*P* = 0.0106; Fig. 3c). Conversely, there was little difference in the expression level of GLO1 between WT ND and WT HFD mice, and only a slight decrease in the levels between ApoE^−/−^ HFD and WT mice.

### Lipid loading targeted glyoxalase 1 for proteasomal degradation with concomitant elevation of intra- and extracellular methylglyoxal (MG)

**Fig. 4:**
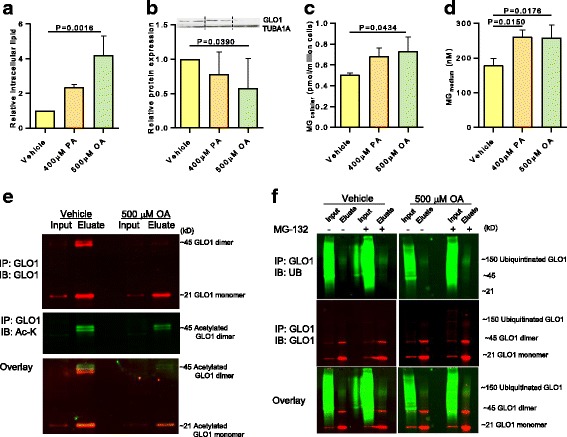
Dysregulation of GLO1 in response to lipid loading. HepG2 cells were treated with vehicle, palmitic acid (PA) or oleic acid (OA) for 24 h. **a** Intracellular lipid accumulation measured by Nile Red assay, **b** Immunoblotting and quantification of GLO1 expression relative to TUBA1A, **c** Cellular methylglyoxal (MG) levels, and **d** Medium MG levels from vehicle or fatty acid treated HepG2 cells. Data are mean ± SD, n = 3–4 independent experiments. **e** Acetylation of endogenous GLO1 immunoprecipitated (IP) from extracts of vehicle or OA treated HepG2 cells with a rabbit anti-GLO1 antibody and immunoblotted (IB) with rat anti-GLO1 and mouse anti-acetylated lysine. Top panel in red is GLO1 monomer (~21kD) and dimer (~ 45) detected in immunoprecitates of GLO1; less GLO1 dimer is immunoprecipitated from OA treated cells. Middle panel in green is detection of the GLO1 dimer with an anti-acetyl-lysine antibody, with less observed in the GLO1 immunoprecipitates of OA treated cells. Bottom panel is overlay. **f** Ubiquitination of endogenous GLO1. Top panel shows ubiquitin detection between 100-150kD in green, in immunoprecipitates of endogenous GLO1; addition of the proteasome inhibitor MG-132 increases GLO1 detection, particularly in OA treated cells. Middle panel shows GLO1 detection in red; very faint banding in the 150 K range only detectable in the eluates from OA and MG-132 co-treated cells. Bottom panel is overlay

Having established a relationship between a high fat diet and decreased expression of GLO1 in the murine model, we explored the potential underlying regulatory mechanism in liver cells in vitro using the hepatoma cell line HepG2. HepG2 cells treated with fatty acids will accumulate lipid droplets and are considered a suitable in vitro model system for the study of steatosis in human hepatocytes [33, 34]. Measurement of Nile red staining showed that treating HepG2 cells with oleic acid (OA) for 24 h led to a fourfold increase in the level of intracellular lipid (*P* = 0.0016; Fig. 4a). Immunoblotting revealed that this increase was accompanied by a significant decrease in the expression of GLO1 protein (*P* = 0.0390; Fig. 4b). Similar treatment with palmitic acid (PA) only induced a twofold increase in intracellular lipid levels (*P* = 0.0738; Fig. 4a) and a slight decrease in GLO1 protein levels (*P* = 0.3451; Fig. 4b), thus appearing to relate to the amount of lipid accumulated in the cells. Importantly, a functional effect of the decrease in GLO1 protein levels was apparent with an increase in the intracellular levels of the GLO1 substrate, MG in both OA and PA treated cells (Fig. 4c) compared to vehicle controls, as measured by the gold standard stable isotopic dilution LC-MS/MS methodology [35]. This was accompanied by a significant increase in the level of MG in the culture media (OA: *P* = 0.0176; PA: *P* = 0.0150, respectively; Fig. 4d). It is difficult precisely to compare levels/doses of OA and PA between the in vitro and animal models as the animals were ad libitum fed a chow diet containing 21% lard, of which ~ 25% is PA and ~ 45% is OA. Interestingly, Tous and colleagues used 20% palm oil, which is 48% SFA with ~ 44% PA & ~ 40% OA and reported a relatively robust liver phenotype after 10 weeks on diet. However, curiously the pathology appeared to resolve considerably after 16 weeks on diet, perhaps calling into question the utility of these model systems for studying the human NAFLD spectrum.

Functional analyses of our proteomic data had highlighted the annotation “acetylation” as a post-translational modification among the identified proteins. Along with this, global acetylation-site screening data from rats [36] suggested that GLO1 might be a target of regulatory post-translational modification. This was validated by immunoprecipitation and immunoblotting using an antibody against acetylated lysines. Both assays provided evidence that the endogenous GLO1 homodimer, the active form of the enzyme, was acetylated in HepG2 cells. Immunoprecipitates from OA treated cells exhibited lower levels of the GLO1 homodimer compared to untreated vehicle cells (Fig. 4e). Independent confirmation was provided by the MS/MS data which identified the acetylated band as GLO1 (Additional file: Figure S6). GLO1 is recognized to be both acetylated and ubiquitinated at multiple amino acids, which vary slightly between human and mouse (human: K-88-ub, K140-ac, K-140-ub, K148-ac, K-148, K157-ac, K159-ac), and are detailed within the PhosphoSitePlus manually curated knowledgebase [37]. As detailed in Hornbeck et al. 2012, these data come from both journal publications and the CST research group and are based on annotated MS spectra [37]. Although we were able to identify our immunoprecipitated band as GLO1 (Additional file 1: Figure S6), we were not able to quantify this in our iTRAQ experiment. These findings suggested that hyperacetylation targeted the active form of the GLO1 enzyme for degradation, as seen in other metabolic enzymes [38]. We examined this hypothesis further by testing for the presence of ubiquitinated proteins, after applying the proteasomal inhibitor MG-132 prior to immunoprecipitation of GLO1. Immunoblotting showed a marked increase in ubiquitinated proteins in the lysates of both vehicle and OA treated cells (Fig. 4g). Furthermore, immunoprecipitation showed that OA treated cells were clearly polyubiquitinated and that inhibition of the proteasome resulted in a 50% increase in the GLO1 homodimer (Fig. 4g).

In summary, these data demonstrated that elevated intracellular lipid levels in hepatic cells induced hyperacetylation, followed by ubiquitination and ultimately degradation of GLO1, with a concomitant accumulation of extracellular levels of MG.

### Adult patients with NAFLD exhibit altered levels of serum MG-derived hydroimidazolone (MG-H1)

The in vitro data had implied an association between the degradation of GLO1 protein in hepatocytes and an increase in extracellular MG in response to high levels of intracellular lipid. MG is a major source of AGEs; therefore, to assess the relevance of this finding in human NAFLD, we investigated the levels of MG-H1, the most abundant MG-derived AGE, and the soluble receptor for AGEs (sRAGE) in serum collected from a cohort of biopsy-confirmed adults with NAFLD (Table 1).

**Table 1:** Patient Characteristics^1^

*N*	62
Age (years)	47.4 ± 1.36
Male (%)	51
BMI (kg/m^2^)	36.8 ± 0.96
BMI < 25 (%)	1.6
BMI 25 ≤ x < 30 (%)	17.7
BMI ≥30 (%)	80.6
NAS^2^ (0–8)	4 (3–5.25)
Steatosis (0–3)	2 (1–3)
Inflammation (0–3)	1 (0–1)
Ballooning (0–2)	1 (0–2)
Fibrosis^2^ (0–4)	1 (0–1.25)
MG-H1 (pmol/mg protein)	0.902 ± 0.050
sRAGE (pg/ml)	565.6 ± 19.5
ALT (IU)	66.35 ± 6.82
ALP (IU)	85.61 ± 3.76
Bilirubin (μmol/L)	11.79 ± 1.4
Cholesterol (mmol/L)	5.08 ± 0.19
TAG (mmol/L)	2.07 ± 0.23
T2D (%)	19.4
HBP (%)	35.5

^1^Mean ± SEM except as noted; ^**2**^median with 25% - 75% percentile. Abbreviations: *BMI*, body mass index; *NAS*, NAFLD activity score; *MG-H1*, methylglyoxal-hydroimidazolone 1; sRAGE, soluble receptor for advanced glycation endproducts; *ALT*, alanine aminotransferases; *ALP*, alkaline phosphatase; *TAG*, triacylglycerol; *T2D*, type 2 diabetes; HBP, high blood pressure.

Univariate analysis of competitive ELISA data showed that the MG-H1 residue content of serum protein was significantly correlated with both ALT (*r* = − 0.375; *P* = − 0.0034) and ballooning (*r* = − 0.351; *P* = 0.0064; Table 2). However, there was no relationship between MG-H1 residue content and inflammation or NAFLD activity score (NAS; Table 2).

**Table 2:** Correlation of MG-H1 with clinical features of NAFLD

Variables	*r*	*P*
MG-H1 versus NAS	−0.205	0.1203
MG-H1 versus inflammation	−0.204	0.1215
MG-H1 versus ballooning	−0.351	0.0064
MG-H1 versus ALT	−0.375	0.0034
MG-H1 versus BMI	0.520	< 0.0001

Pearson or Spearman correlation was used to analyse the relationship between variables. Abbreviations: *MG-H1*, methylglyoxal-hydroimidazolone 1; *NAS*, NAFLD activity score; *ALT*, alanine aminotransferase; *BMI*, body mass index

**Fig. 5:**
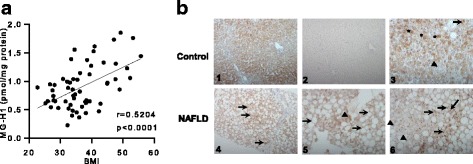
Serum MG-H1 levels and altered GLO1 expression in NAFLD patients. **a** Correlation of MG-H1 with body mass index (BMI) in adult NAFLD patients (*n* = 59). **b** GLO1 immunohistochemistry in a non-NAFLD control case (panels 1,2,3) and in the biopsy specimen of a NAFLD patient (panels 4,5,6). 1: Low magnification reveals expression of GLO1 in hepatocyte cytoplasm without a particular zonal distribution. 2: Low magnification of a negative control section without the primary antibody. 3: High magnification shows cytoplasmic staining (asterisks) and focal nuclear staining (arrow) in hepatocytes. Not all hepatocytes appear to express GLO1 (black triangle). 4: Low magnification reveals bridging fibrosis and severe steatosis with retention of GLO1 staining in non-steatotic foci (arrows); 5: high magnification shows cytoplasmic staining of GLO1 in non-steatotic hepatocytes (black triangle). A peripheral staining rim is noted around large steatotic droplet, and expression of GLO1 in marginalised cytoplasm cannot be excluded (arrows). 6: High magnification reveals nuclear staining (arrows) of GLO1 in many, but not all (triangles), hepatocyte nuclei

The strongest correlation was found between the MG-H1 residue content in serum protein and body mass index (BMI; *r* = 0.520, *P* < 0.0001; Fig. 5a). Furthermore, when all variables were examined by multiple linear regression, a model could be fitted whereby BMI predicted MG-H1 levels. Although univariate analysis had shown that the median MG-H1 residue content appeared to be lower in serum protein from patients with NASH relative to those with simple steatosis, this was not significant and there was no difference in the level of sRAGE, the soluble receptor for AGEs, between patients with NASH and those with simple steatosis (Additional file 1: Figure S7). In addition, the correlation analysis found no associations between sRAGE and any of the tested clinical features of NAFLD (Additional file 1: Table S3).

### Expression of glyoxalase 1 was altered in paediatric patients with NAFLD

While diagnosis of primary NAFLD in adults may be confounded by secondary causes (e.g., severe weight loss, drug, and alcohol consumption), paediatric NAFLD is considered an excellent model to elucidate the factual origins of disease [39]. We therefore chose to examine GLO1 expression by comparing liver biopsies between nine paediatric patients with NAFLD and three non-NAFLD controls. Histological review of the nine NAFLD liver biopsy samples noted various degrees of disease severity, from mild steatosis with mild or no fibrosis, to marked steatosis with mild or bridging fibrosis. NAS scores of the patient samples ranged from 2 to 5 and fibrosis was scored F1 in 4, F2 in 1, and F3 in 4 biopsies (Additional file 1: Table S4); whereas, histological review of the three control cases revealed minimal and non-specific changes.

GLO1 expression in the control cases was diffuse without particular zonality, and the staining was predominantly cytoplasmic with minimal focal staining suggestive of nuclear expression (Fig. 5b, panels 1 and 3). In comparison, the staining patterns in the biopsy sections from the NAFLD cases differed from the control cases, and numerous hepatocytes, with or without cytoplasmic changes, showed nuclear staining for GLO1 (Fig. 5b panels 4–6). In general, non-steatotic non-ballooned hepatocytes retained GLO1 cytoplasmic expression. Whereas in steatotic hepatocytes, characterized by single large intracytoplasmic vacuoles, the staining could not be easily assessed and it appeared to be confined to the peripheral part of cytoplasm, possibly marginalized by the large steatotic droplet. As such, the role of immunohistochemistry as a tool for investigating cytoplasmic expression of GLO1 may need to be questioned in the context of NAFLD.

## Discussion

GLO1 deficiency is recognized as contributing to the pathogenesis of obesity and diabetic complications. Metabolic syndrome and obesity have also been linked to the development and progression of NAFLD. However, the role of GLO1 in the pathogenesis of NAFLD has not been established and the involvement of the glyoxalase system in NAFLD is relatively unexplored.

The biochemical function of GLO1 is the glutathione-dependent metabolism of the reactive dicarbonyl metabolites, glyoxal and MG, to glycolate and D-lactate respectively, in order to prevent irreversible protein glycation and the accumulation of AGEs. A large body of work by Rabbani and Thornalley over the last two decades has demonstrated that disturbance of the glyoxalase system plays a central role in the development of the vascular complications associated with diabetes and obesity [19]. In the context of diabetes, hyperglycemia drives an increase in glycolytic flux thereby increasing MG levels and the formation of AGEs. Parallel downregulation of GLO1 expression further exacerbates the formation of MG modified proteins leading to diabetic nephropathy and neuropathy [40, 41]. It has been claimed that the downregulation of GLO1 expression is a result of signalling through the multi-ligand receptor for AGEs (RAGE) [42]. The AGE N^ε^-(carboxymethyl)-lysine (CML), derived from oxidative degradation of fructosamine and glyoxal has been shown to be increased in fatty liver, and associated with the severity of steatosis and inflammation [22]. Glyoxal is a substrate for GLO1 and hence a decrease in GLO1 activity will contribute to an increase in CML formation. Taken together with previous reports of decreased hepatic formation of S-D-lactoylglutathione [21] and increased MG-H1 residue content of hepatic plasma protein [43] in experimental models of NASH, this finding suggested that increased GLO1 proteolysis in the liver in obese subjects leads to the increased dicarbonyl glycation implicated in NASH. High fructose fed rats were reported to have decreased GLO1 activity [20], and in a recent metabolomics study, LDLR^−/−^ mice with high fat diet-induced NASH showed an 83% decrease in hepatic content of S-D-lactoyl-glutathione, consistent with a marked decrease in GLO1 activity [21]. Separately, hepatic GLO1 expression was decreased in a murine model of hyperhomocysteinemia-induced fatty liver [44]. In agreement with these reports, we had independently identified GLO1 as being downregulated in fatty acid treated HuH7 cells in a pilot proteomic experiment [30] and report here novel data showing alterations in GLO1 in both animals with early signs of diet-induced liver injury and paediatric patients with NAFLD.

**Fig. 6:**
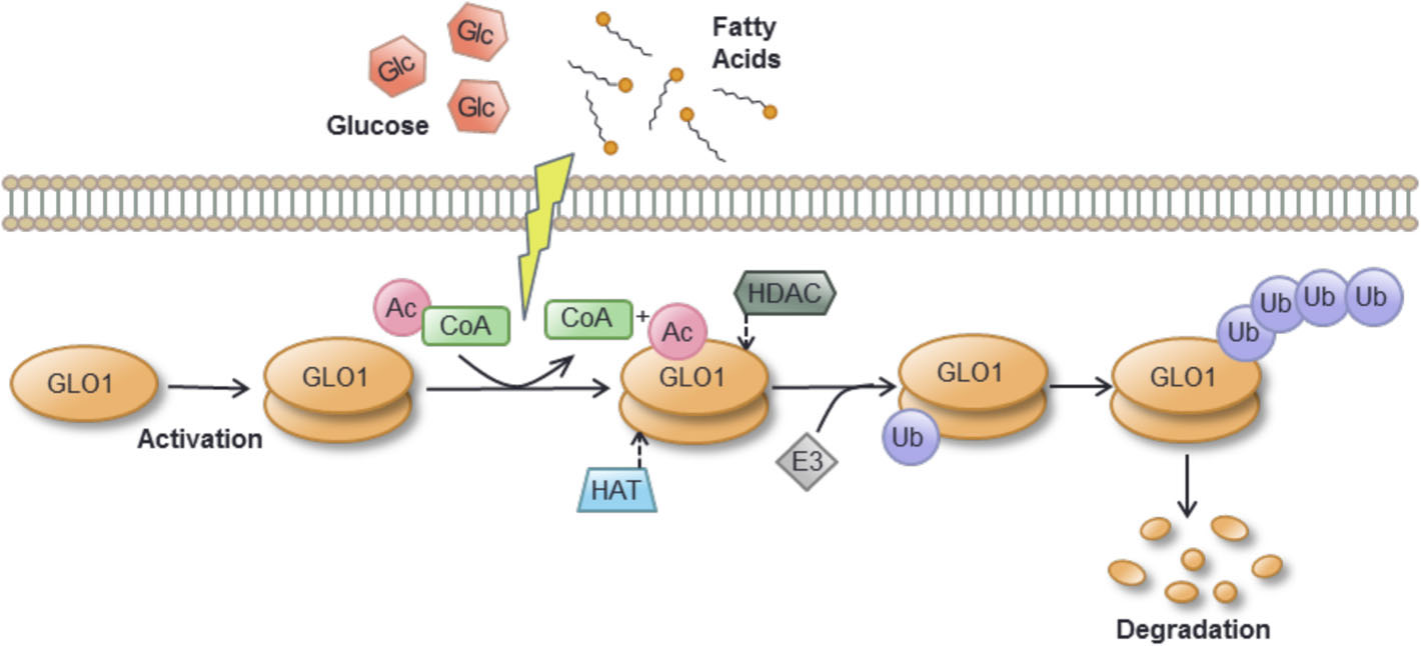
Working model for post-translational regulation of GLO1 expression in NAFLD

The regulatory scope of protein acetylation is increasingly being recognized, largely as a result of advances in proteomic technology [38, 45, 46]; and evidence has emerged that the majority of enzymes involved in intermediary liver metabolism are dynamically acetylated in response to extracellular nutrient availability [38]. Proteome-wide analyses of tissue-specific acetylation sites in rat identified the lysine K148 of GLO1 as highly acetylated in liver, kidney, stomach and testis [36]; and another study demonstrated that fatty liver was associated with protein hyperacetylation due to the reduced activity of the mitochondrial SIRT3 deacetylase [47]. Consistent with these data, we found that GLO1 was acetylated in response to lipid loading in HepG2 cells. Furthermore, our results have provided novel evidence that hyperacetylation, ubquitinylation and proteasomal targeting of endogenous GLO1 are involved in the downregulation of GLO1 in response to fatty acids and lipid loading. The resulting decrease in expression of GLO1 protein led to an increase in both intra- and extra-cellular levels of MG. Based on these observations, we have proposed a model for the post-translational regulation of GLO1 expression in response to shifts in cellular nutrient flux (Fig. 6).

MG-derived AGEs have been studied in cirrhosis in both rats and humans [48, 49], but not in the pathogenesis of NAFLD. In humans, a threefold increase in the level of MG-H1 residue content in plasma protein in cirrhosis was linked to a decrease in the concentration of plasma albumin and catabolism [49]. Separately, patients with NASH were reported to have elevated serum levels of glyceraldehyde-derived AGE. However, it is unclear if serum protein AGE residue content, AGE free adducts or both contributed to the response, and the epitope specificity of the antibody used is uncertain [50]. sRAGE has been shown to be significantly lower in obese children with steatosis as well as lower in adult NASH patients compared to healthy controls [51, 52]. However, no significant associations were found between the level of sRAGE and clinical features of NAFLD in our cohort. This may be attributed to the low number of participants or differences in the commercial enzyme immunoassays used.

To our knowledge, there have been no previous reports of MG-H1 levels in patients with NAFLD. Although univariate analysis had suggested that serum MG-H1 levels in our cohort of adult patients with NAFLD, were correlated with ALT and ballooning (hepatocyte injury and feature of NASH), multiple linear regression indicated that the MG-H1 residue content of serum was not a reliable predictor of NAFLD severity, but was significantly positively correlated with BMI (*P* < 0.0001). This relationship was of interest as it implied that BMI may be a predictor of MG-H1 residue content in plasma protein, linking serum MG-H1 levels with obesity, a major risk factor for NAFLD. This contrasts with a recent study in subjects without NAFLD that compared 18 non-obese with 29 obese adults. Although MG levels were found elevated by a ~ 35% in the obese subjects, there was no difference in MG-H1 levels; a result the authors suggest could relate to decreased residence time of albumin from plasma to interstitial fluid in obesity [18]. However, it has been observed that plasma protein AGE content may not be reflective of AGE tissue exposure. In a recent trial that supplemented obese subjects with a formulation of dietary bioactives that induce GLO1, both plasma MG and the endogenous formation of MG-H1 adducts flux were decreased without a change in plasma protein MG-H1 [53].

Quantitative analysis of MG-H1 residue content of rat liver protein suggests relatively high steady-state levels are maintained in health, 3.3 mmol/mol arginine; therefore, GLO1 deficiency in NAFLD may overwhelm the hepatic capacity for removal of MG-H1-modified proteins contributing to NAFLD progression as suggested by our results. This possibility merits further research particularly in light of results from the aforementioned randomized trial showing that supplementation of GLO1 inducers (the dietary bioactives trans-resveratrol and hesperetin) for 8 weeks decreased fasting and postprandial glucose levels and increased insulin sensitivity [53]. Validation of these concepts in NAFLD will require future prospective intervention studies in larger cohorts, ideally using gold-standard LC-MS/MS methodology to comprehensively assess plasma AGEs and endogenous flux in NAFLD longitudinally, as previously demonstrated in type 1 diabetes [54].

## Conclusions

In summary, from a hypothesis-generating proteomic screen of hepatic proteins altered in a murine model, we identified GLO1 protein as downregulated and have observed an altered pattern of GLO1 expression in liver biopsies from paediatric patients with NAFLD. We also found a strong association between elevated levels of serum MG-H1 and obesity in adults with NAFLD. We have observed that GLO1 is hyperacetylated, ubiquitinated and degraded in response to an accumulation of intracellular lipid, with metabolic consequences for the hepatocyte, providing novel insights into the mechanism underlying the response of GLO1 to shifts in cellular nutrient content. These data in total suggest that GLO1 dysregulation may play a role in NAFLD pathogenesis**.**

## Methods

### Clinical samples

In accordance with Helsinki guidelines, informed written consent was obtained from adult patients and the caregivers of the paediatric patients participating in this study. The study was approved by the Tayside Medical Ethics Committee and the King’s College Hospital NHS Foundation Trust LEC (REC number 09/H0808/15).

A total of 62 adult patients with biopsy-proven NAFLD and negative liver screens (negative for viral hepatitis, autoimmune liver disease, haemochromatosis and alcoholic liver disease) were included. Subjects were excluded if their alcohol intakes were > 21 units/week (male) or > 14 units/week (female). Serum samples were collected from patients within three months of liver biopsy and then centrifuged at 10,000×g for 10 min before being stored in 100 μl aliquots at 80 °C within 4 h of collection.

Liver biopsies from nine paediatric patients with NAFLD and three control patients were used in this study. Liver biopsy was undertaken as part of routine clinical care and undertaken by a trained paediatric hepatologist using the Menghini technique. None of the children with NAFLD displayed other forms of liver disease, including Wilson’s disease, viral hepatitis and inborn errors of metabolism. Due to ethical constraints, the control biopsies included incidental or routine biopsies collected from children with non-NAFLD diseases. These included a child with autoimmune pancreatitis, a child exhibiting developmental delay (underwent biopsy for respiratory chain analysis as part of metabolic work up), and a child with Hepatitis C. Control biopsies were histologically confirmed as normal by an experienced hepato-histopathologist, and liver function tests were entirely normal.

### Animal experiments

All animal care and experimental procedures complied with the Animals (Scientific Procedures) Act 1986, and were approved by the University of Surrey Animal Ethics Committee. Animals received humane care and experiments were designed to minimise the number of animals used. Male mice 6–8 weeks old were purchased from Charles Rivers, UK (apolipoprotein E knockout [ApoE^−/−^], strain B6.129P2-Apoe^tm1Unc^/J on a C57BL/6 background, and wild type [WT]).

The mice were housed in microisolation cages at constant temperature on a standard 12-h dark/light cycle. They were fed ad libitum for up to 16 weeks with either a normal diet (ND; 2.5% fat, 13% protein, 84.5% starch-based carbohydrate; Special Diets Services, UK) or a high fat diet (HFD; 21% fat (lard), 0.17% cholesterol, 18% protein, 60.5% sucrose-based carbohydrate; Teklad, UK). This atherogenic HFD has been shown to induce NASH in ApoE^−/−^ mice [31]. The mice were sacrificed at 4, 8, 12 and 16 weeks (*n* = 3 from each group at each time point). Blood was collected every four weeks from start of diet from alternate lateral tail veins of animals under restraint. Animals were euthanized by intraperitoneal injection of sodium pentobarbital (200 mg/kg).

On the day of termination, blood was collected from the hepatic portal vein and the heart and liver were excised and perfused with phosphate-buffered saline (PBS) until the majority of the blood was cleared. The heart and right liver lobe were immersed in phosphate-buffered formalin for histological examination while the left liver lobe was snap frozen in liquid nitrogen and stored at − 80 °C for subsequent use in proteomic-related studies. Blood samples were centrifuged at 13,000×g for 23 min for plasma collection. Total cholesterol, HDL cholesterol and TAG measurements were done using the commercially available enzymatic kits (Randox, Northern Ireland) and an automated analyzer, (iLab 650; Instrumentation Laboratory, U.K.) according to manufacturer’s protocol. The non-HDL cholesterol (VLDL mainly, and LDL) was determined by subtracting the HDL cholesterol. In order to measure liver TAG, liver tissue was homogenized in chloroform/methanol (2:1, *v*/v) and lipid was extracted using the Folch method [55], evaporated under N_2_ and re-suspended in 2% (v/v) Triton X-100. TAG concentrations were measured using a commercial assay (Sigma Aldrich, UK).

### Proteomics

#### Protein extraction

Samples were prepared and labelled with iTRAQ reagents as has been described in detail previously [56]. In brief, murine liver tissue was homogenised in ice-cold buffer containing 20 mM Hepes, 1 mM ethylenediaminetetraacetic acid, 300 mM mannitol and protease inhibitors (HEM buffer), then samples were centrifuged at 2000×g for 10 min at 4 °C. Supernatants were then centrifuged at 100,000×g for 30 min. The membrane-enriched pellet was resuspended in fresh HEM buffer, while the cytosolic-enriched supernatant was concentrated by centrifuging for 1 h at 4000×g through an Amicon ultra-15 centrifugal filter device (3 kDa MWCO; Millipore, UK). Sample concentrations were first estimated by the Bradford assay (Sigma, UK). Samples were desalted thereafter using Zeba columns (7 kDa MWCO; Pierce, UK) and protein concentration was measured by BCA assay (Pierce UK).

#### iTRAQ labelling and nLC-MS/MS

100 μg of cytosolic- and membrane-enriched liver protein samples from each animal were digested using mass spectrometry grade trypsin gold (1:10, trypsin:protein ratio; Promega, UK). The resulted peptides were labelled with iTRAQ reagents (AB Sciex, UK) according to the manufacturer’s protocol, with 114, 115, 116, and 117 tags corresponding to WT ND, WT HFD, ApoE^−/−^ ND and ApoE^−/−^ HFD groups (*n* = 3 biological replicates), respectively. Labelled peptides were prefractionated, (24 fractions) by isoelectric point using the 3100 OFFGEL fractionator (Agilent Technologies, UK) following the manufacturer’s instructions, then dried under vacuum centrifugation (Concentrator 5301; Eppendorf, UK) and resuspended in 0.1% (v/v) formic acid. All LC/MS experiments were performed on a 1200 Series HPLC-Chip interfaced to a 6520 accurate-mass quadrupole time-of-flight LC/MS (Agilent Technologies, UK). The HPLC-Chip configuration consisted of a 160 nL enrichment column and a 150 mm × 75 μm analytical column (Zorbax 300SBC18). Mobile phases were buffer A: 0.1% formic acid in LC/MS grade water, and buffer B: 95% acetonitrile with 0.1% (v/v) formic acid in LC/MS grade water. Sample loading onto the enrichment column was done at 4% B. The gradient used for the analytical column began at 4% B, raised to 25% B at 21 min, 45% B at 22.8 min, 65% at 28.8 min, 90% at 29.6 min, maintained at 90% B until 30.4 min and then brought back to 4% B at 31.8 min. The column was equilibrated for 10 min before subsequent runs. Samples were loaded at 2.7 μL/min flow rate and eluted at 320 nL/min. The LC/MS was operated in high resolution (4 GHz) positive ion mode and the MS source conditions were source temperature: 325 °C; capillary voltage: 1850 V; fragmentor voltage: 170 V; and drying gas flow rate: 3 L/min. Data was acquired between *m/z* 50–3200 at a scan rate of 1 spectra/s for all samples in the profiling experiments. In the targeted MS/MS experiment, data was acquired from 50 to 3200 *m/z* with an acquisition rate of 4 spectra/s in MS mode and *m/z* region 50–3200 with an acquisition rate of 5 spectra/s in MS/MS mode. Complete system control was achieved using Agilent MassHunter data acquisition software (B.03.00).

#### Data analysis

Tandem mass spectra were extracted and charge state deconvoluted by Mass Hunter version B.03.00 (Agilent Technologies, USA), while Spectrum Mill and X!Tandem (The GPM, thegpm.org; version 2007.01.01.1) were used for protein identification. Deisotoping was not performed. Both Spectrum Mill and X!Tandem were set up to search a subset of the Swiss-Prot database (Release-2011_07) (UniProtKB, Swiss-Prot) selected for *Mus musculus* and assuming digestion with trypsin. The search was performed with a fragment ion mass tolerance of 50 ppm and a parent ion tolerance of 20 ppm. Oxidation of methionine, methyl methanethiosulfonate of cysteine and ABSciexiTRAQ™(0.5 M Triethylammonium bicarbonate, 2% SDS, 50 mM Tris2-carboxyethyl-phosphine hydrochloride and 200 mM MMTS) multiplexed quantitation chemistry of lysine and the n-terminus were specified in X!Tandem as variable modifications. Scaffold Q+ (version Scaffold_4.8.4, Proteome Software Inc., USA) was used for final protein identification and iTRAQ quantitation. Thresholds of 95% and 99% probability were used for peptide and protein identifications in Scaffold respectively. In addition, protein identification required at least 2 unique identified peptides. Protein probabilities were assigned by the Protein Prophet algorithm [57] and the calculated FDR for protein identification was less than 0.05%. Proteins that contained similar peptides and could not be differentiated based on MS/MS analysis alone were grouped to satisfy the principles of parsimony. Acquired intensities in the experiment were globally normalized across all acquisition runs. Individual quantitative samples were normalized within each acquisition run. Intensities for each peptide identification were normalized within the assigned protein. The reference channels were normalized to produce a 1:1 fold change. All normalization calculations were performed using averages to multiplicatively normalize data. Differentially expressed proteins were determined using both randomised permutation and Kruskal-Wallis tests with Benjamini-Hochberg FDR for multiple testing corrections. GO annotations were assigned by Scaffold_4.8.4 Q+. The DAVID version 6.8 [23], Reactome pathway version 63 [25], and KEGG pathway release 80 [27] knowledgebases were used alongside their, and others tools, including the enrichment analysis tool Enrichr [26] for bioinformatics analyses.

#### Data repository

The MS proteomics data have been deposited to the ProteomeXchange Consortium (http://proteomecentral.proteomexchange.org) via the PRIDE partner repository [58] with the dataset identifier PXD001442.

### Histopathology and immunohistochemical analyses

#### Murine liver and heart tissue

Right liver lobes and hearts were fixed in 10% phosphate-buffered formalin, embedded in fibrowax (BDH, UK), sliced into sections (4 μm thick) and stained with H&E or Masson’s trichrome. The liver sections were blindly evaluated and scored by a qualified veterinary pathologist (FJS) for the degree of non-alcoholic steatohepatitis, as described by Kleiner [59]. The heart sections were examined by image analysis software (ImageJ) [60] for quantification of atherosclerotic plaques in the proximal aortic sinuses.

Immunolabelling of GLO1 was performed by the avidin-biotin complex method (ABC Vector Elite; Vector laboratories, USA). Briefly, 4-μm thick sections were dewaxed and rehydrated. Endogenous peroxidase inhibition was carried out in 3% H_2_O_2_ in methanol for 30 min using a Sakura Tissue Tek DRS 2000 slide autostainer, and antigen retrieval was performed by microwaving the sections in 0.1 M citric acid buffer, pH 6.0 (Sigma Aldrich, UK) containing 0.01% Tween 20 (Fisher Scientific; UK). Slides were cooled to room temperature (RT) for 30 min, mounted in a Sequenza Immunostaining Centre (Shandon Scientific, UK) and washed with Tris buffered saline (TBS; 0.005 M, pH 7.6; Sigma-Aldrich, UK). Slides were then incubated for 30 min at RT with 190 μL/slide blocking solution before being incubated with polyclonal rabbit anti-GLO1 primary antibody (1:100; ab96032-Abcam) for 1 h at RT. The slides were incubated with a biotinylated secondary antibody (1:400; Vector Laboratories; UK) for 30 min at RT followed by 30 min with avidin-biotin complex. Labelling was performed using 3,30-diaminobenzidine tetrahydrochloride (DAB; Sigma-Aldrich, UK). Sections were counterstained with Mayer’s haematoxylin, dehydrated and mounted using the slide autostainer. Images were taken under light microscopy and analysed by image analysis software (Leica DMLB and Leica QWin V3; Leica Microsystems GmbH, Germany). Controls were prepared by labelling serial sections with isotype control (rabbit IgG).

#### Human liver biopsy samples

Needle biopsy specimens collected from paediatric patients were fixed in formalin, embedded in paraffin and stained for routine histological diagnosis (H&E, reticulin, diastase PAS, Perls’ and Orcein). Sections (4 μm thick) were reviewed by a hepatohistopathologist (AQ) blinded to clinical details. Semi-quantitative analysis was performed using the Brunt/Keliner score method. Qualitative examination of sections for GLO1 immunostaining (described in detail above) was performed under an Olympus BX51 light microscope. The staining patterns were assessed with reference to zonality (distribution in relation to lobular or acinar zones) and intracellular distribution (membranous, cytoplasmic or nuclear).

### Cell culture

HepG2 cells were obtained from the European Collection of Cell Cultures and routinely cultured in Dulbecco’s modified Eagle’s medium (DMEM) containing 5.55 mM glucose, 10% fetal bovine serum, 1% nonessential amino acids, 2 mM L-glutamine and 100UI/mL penicillin and streptomycin (all reagents; Lonza, UK). Cells were seeded at a density of 30,000/cm^2^ and cultured for three days prior to treatment. Palmitic acid (PA; Sigma, UK) and oleic acid (OA; Sigma, UK) dissolved in dimethyl sulfoxide (DMSO; Sigma, UK) were complexed with fatty acid free bovine serum albumin (FAF-BSA; Sigma, UK) for 1 h at 37 °C with mixing every 10 min prior to sterile filtration and delivery in serum-free media for a 24 h treatment period; DMSO in FAF-BSA was used as a vehicle control. Cells were harvested by trypsinization and counted on an automated cell counter (T20; Biorad, UK). For measurement of intracellular lipid, 500,000 cells were incubated with 1 μM Nile red (Sigma, UK) in PBS for 10 min at 37 °C. The cells were centrifuged at 800×g for 10 min, the supernatant was discarded and the cell pellet was resuspended in PBS. Intracellular lipid levels were determined by measuring fluorescence using a FLUOstar OMEGA plate reader (BMG LABTECH Ltd., UK) at an excitation of 485-12 nm with a 520 nm emission filter. MG content in cells and culture media was measured by stable isotopic dilution LC-MS/MS [35].

### Immunoblotting

Mouse liver and HepG2 cells were lysed in radioimmunoprecipitation buffer (Sigma, UK) with a protease inhibitor cocktail (Roche, UK). Mouse liver extracts were taken to a final concentration of 10 mM MgCl_2_, 10 mM CaCl_2_ and DNase treated with 50 units of DNase-I (Promega, UK) at 37 °C for 2 h. Subsequent heat inactivation was performed at 65 °C for 30 min. Protein concentration was determined by bicinchoninic acid (BCA) assay (Pierce, UK). Protein lysates were resolved by SDS-PAGE and transferred to polyvinylidene difluoride (PVDF) membranes (Millipore, UK). Membranes were blocked in 5% (*w*/*v*) milk/TBST for 1 h prior to incubation with primary antibody in TBS-Tween20 (T) for 12–16 h at 4 °C. The following antibodies were used: anti-L-FABP (1:4000; ab7847; Abcam, UK); anti-COXIV (1:1000; ab16056; Abcam, UK) and anti-TUBA1A (1:10,000; ab7291; Abcam, UK). Anti-GLO1 (1:1000; ab96032; Abcam, UK) was used for mouse liver samples and anti-GLO1 (1:4000; SAB4200193; Sigma UK) was used for HepG2 extracts. The membranes were washed in TBST and incubated with HRP-conjugated secondary antibodies (Sigma, UK) for 2 h or fluorescently-tagged antibodies (LICOR Biosciences, UK) for 1 h. Proteins were either visualized by enhanced chemiluminescence (Pierce, UK) and exposed to Hyperfilm™ (GE Healthcare, UK), or by infrared fluorescence using a LI-COR Odyssey imaging system (LI-COR Biosciences, UK). Densitometry was performed using ImageJ [60] or Image Studio (LI-COR Biosciences, UK) software.

### Immunoprecipitation of glyoxalase 1

Immunoprecipitation of GLO1 was performed by crosslinking 50 μl of rabbit polyclonal anti-GLO1 (H00002739-D01; Abnova, Taiwan) to protein A/G agarose columns (Pierce, UK) according to the manufacturer’s protocol. Protein lysates from vehicle and fatty acid-treated cells were pre-cleared by mixing 1 mg lysate with control agarose resin for 1 h at 4 °C on a rotator, followed by antibody cross-linked resin overnight at 4 °C. Cleared lysate was eluted according to the manufacturer’s protocol. The immunoprecipitates were resolved by 10% SDS-PAGE and visualized by Coomassie staining (Sigma, UK). Immunoblotting was carried out using rat anti-GLO1 (1:4000; SAB4200193; Sigma UK), mouse anti-acetylated lysine (1:1000; 9681; Cell Signaling Technology, UK) and mouse anti-ubiquitinated proteins (1:1000; 04–263, Millipore, UK). For MS confirmation of GLO1 detection, bands were excised, chopped into 1mm^2^ pieces and destained in 200 mM NH_4_HCO_3_ in 40% (*v*/v) acetonitrile. They were washed in 50 mM NH_4_HCO_3_, dehydrated with acetonitrile and subjected to trypsin proteolysis overnight at 37 °C using 10 ng/ml trypsin in 50 mM ammonium bicarbonate. Any peptides remaining in the excised gel pieces in the supernatant were extracted by sonication for 10 min in 50 μL 50:50 acetonitrile:0.1% v/v formic acid. The supernatants were combined, dried and reconstituted in 3% acetonitrile:97% 0.1% formic acid in water. Each digested sample was loaded onto a 15 cm × 75μmi.d.PepMap, C18, 3 μm column (LC-Packings) using a U3000 HPLC system (Thermo, Hemel Hempstead, UK). HPLC buffers, A and B, consisted of 3% (v/v) acetonitrile:0.1% (v/v) formic acid and 97% (v/v) acetonitrile:0.1% (v/v) formic acid, respectively. Samples were desalted online prior to separation using a micro pre-column cartridge (5 mm × 300μmi.d.). The washing solvent consisted of 3% (v/v) acetonitrile:0.1% (v/v) trifluoroacetic acid delivered at a flow rate of 30 μL/min. Peptides were separated over a gradient of 5% Buffer B to 35% Buffer B over 40 min at a flow rate of 300 nL/min.

### MG-H1 and sRAGE measurements

MG-H1 content in serum samples was measured by competitive ELISA (Cell Biolabs, UK) and expressed relative to serum protein content determined by BCA assay. sRAGE was measured by sandwich ELISA (Cell Biolabs). ELISA procedures were performed according the manufacture’s protocols.

### Statistical analysis

Experiments were performed in triplicate. Animal and cell results were expressed as mean ± SEM and were analysed by two-way or one-way ANOVA with Tukey’s or Dunnetts post hoc test as appropriate. Human data were tested for normality using the D’Agostino-Pearson omnibus test and were analysed by ANOVA/Kruskal Wallis with Tukey’s/Dunn’s multiple comparison post hoc test and Spearman’s/Pearson’s correlation, as appropriate. For multiple linear regression, the missing values were imputed using multiple imputation technique. Model selection procedure was carried out using backward stepwise regression. The model with the lowest AIC value was considered the best fit. A *P*-value < 0.05 was considered statistically significant.

## Additional files

Additional file 1: Additional Figures and Tables. (PDF 1738 kb)

Additional file 2: Peptide Lists. (XLSX 1682 kb)

Additional file 3: Protein Lists. (XLSX 309 kb)

## Abbreviations

AGEs: Advanced glycation endproducts
ApoE^-/-^: Apolipoprotein E knockout
BMI: Body mass index
DAVID: Database for annotation, visualization and integrated discovery
FASN: Fatty acid synthase
GLO1: Glyoxalase 1
HFD: High fat diet
iTRAQ: Isobaric tags for relative and absolute quantitation
L-FABP: Liver fatty acid binding protein
MG-H1: Methylglyoxal-derived hydroimidazolone 1
NAFLD: Non-alcoholic fatty liver disease
NAS: NAFLD activity score
NASH: Non-alcoholic steatohepatitis
ND: Normal diet
nLC-MS/MS: Nano-liquid chromatography tandem mass spectrometry
RAGE: Receptor for advanced glycation end products.
SP_PIR_KEYWORD: Swiss-Prot and protein information resource keywords
sRAGE: Soluble receptor for advanced glycation endproducts
TAG: Triacylglycerol
WT: Wild type
