# Correction to: Proteomic identification and characterization of hepatic glyoxalase 1 dysregulation in non-alcoholic fatty liver disease

**DOI:** 10.1186/s12953-018-0142-8

**Published:** 2018-06-25

**Authors:** Christos Spanos, Elaina M. Maldonado, Ciarán P. Fisher, Petchpailin Leenutaphong, Ernesto Oviedo-Orta, David Windridge, Francisco J. Salguero, Alexandra Bermúdez-Fajardo, Mark E. Weeks, Caroline Evans, Bernard M. Corfe, Naila Rabbani, Paul J. Thornalley, Michael H. Miller, Huan Wang, John F. Dillon, Alberto Quaglia, Anil Dhawan, Emer Fitzpatrick, J. Bernadette Moore

**Affiliations:** 1 Department of Nutritional Sciences, Faculty of Health and Medical Sciences, University of Surrey, Guildford, Surrey, GU2 7XH UK; 2 Institute of Child Health, University College London, WC1N 1EH, London, UK; 3 Biological and Systems Engineering Group, ChELSI Institute, Department of Chemical and Biological Engineering, University of Sheffield, S1 3JD, Sheffield, UK; 4 Molecular Gastroenterology Research Group, Department of Oncology and Insigneo Institute for in silico Medicine, University of Sheffield, S10 2RX, Sheffield, UK; 5 Clinical Sciences Research Laboratories, Warwick Medical School, University of Warwick, University Hospital, Coventry, CV2 2DX UK; 6 Medical Research Institute, University of Dundee, Ninewells Hospital and Medical School, Dundee, DD1 9SY UK; 7 Paediatric Liver, GI and Nutrition Centre, King’s College London School of Medicine, London, SE5 9RS UK; 8 School of Food Science and Nutrition, University of Leeds, Leeds, LS2 9JT UK

## Correction

Following publication of the original article [1], J. Bernadette Moore noticed that her name was incorrectly listed on PubMed as:

Given name: J.

Surname: Bernadette Moore

This should in fact be:

Given name: J. Bernadette

Surname: Moore
