# Supplementary material for: Proteomic identification and characterization of hepatic glyoxalase 1 dysregulation in non-alcoholic fatty liver disease
Source: Proteome Sci. 2018 Feb 14;16:4. doi: 10.1186/s12953-018-0131-y (PMC5813374; doi:10.1186/s12953-018-0131-y)
Supplement: Supplementary file 1 — Additional Figures and Tables. (PDF 1738 kb) [file 12953_2018_131_MOESM1_ESM.pdf]

SUPPLEMENTARY MATERIAL

Proteomic identification and characterization of hepatic glyoxalase 1 dysregulation in non-alcoholic fatty liver disease

Spanos C, Maldonado EM, Fisher CP, Leenutaphong P, Oviedo-Orta E, Windridge D, Salguero FJ, Bermúdez-Fajardo A, Weeks ME, Evans C, Corfe BM, Rabbani N, Thornalley PJ, Miller MH, Wang H, Dillon JF, Quaglia A, Dhawan A, Fitzpatrick E and Moore JB[1].

[1]Corresponding Author: J. Bernadette Moore j.b.moore@leeds.ac.uk

Table of Contents

Fig. S1. Proteomic workflow. 2

Fig. S2. iTRAQ-based proteomic analysis of murine liver membrane and cytosolic proteins.. 3

Fig. S3. Clustergram of gene names for differentially expressed proteins significantly overrepresented in KEGG pathways. 4

Fig. S4. Proteomic identification of lactoylglutathione lyase (glyoxalase 1; GLO1). 5

Fig. S5. Hepatic fibrosis and heart pathology after 12 or 16 weeks. 6

Fig. S6. Mass spectrometry confirms immunoprecipitated band as GLO1. 7

Fig. S7. Serum levels of MG-H1 and sRAGE in adult NAFLD patients. 8

Table S1. Differentially expressed proteins in liver cytosolic fraction 9

Table S2. Differentially expressed proteins in liver membrane fraction 13

Table S3. Correlation of sRAGE with clinical features of NAFLD 17

Table S4. Histopathological scoring for individual biopsies from 9 pediatric 18

patients with NAFLD 18

Supplementary Figure 1

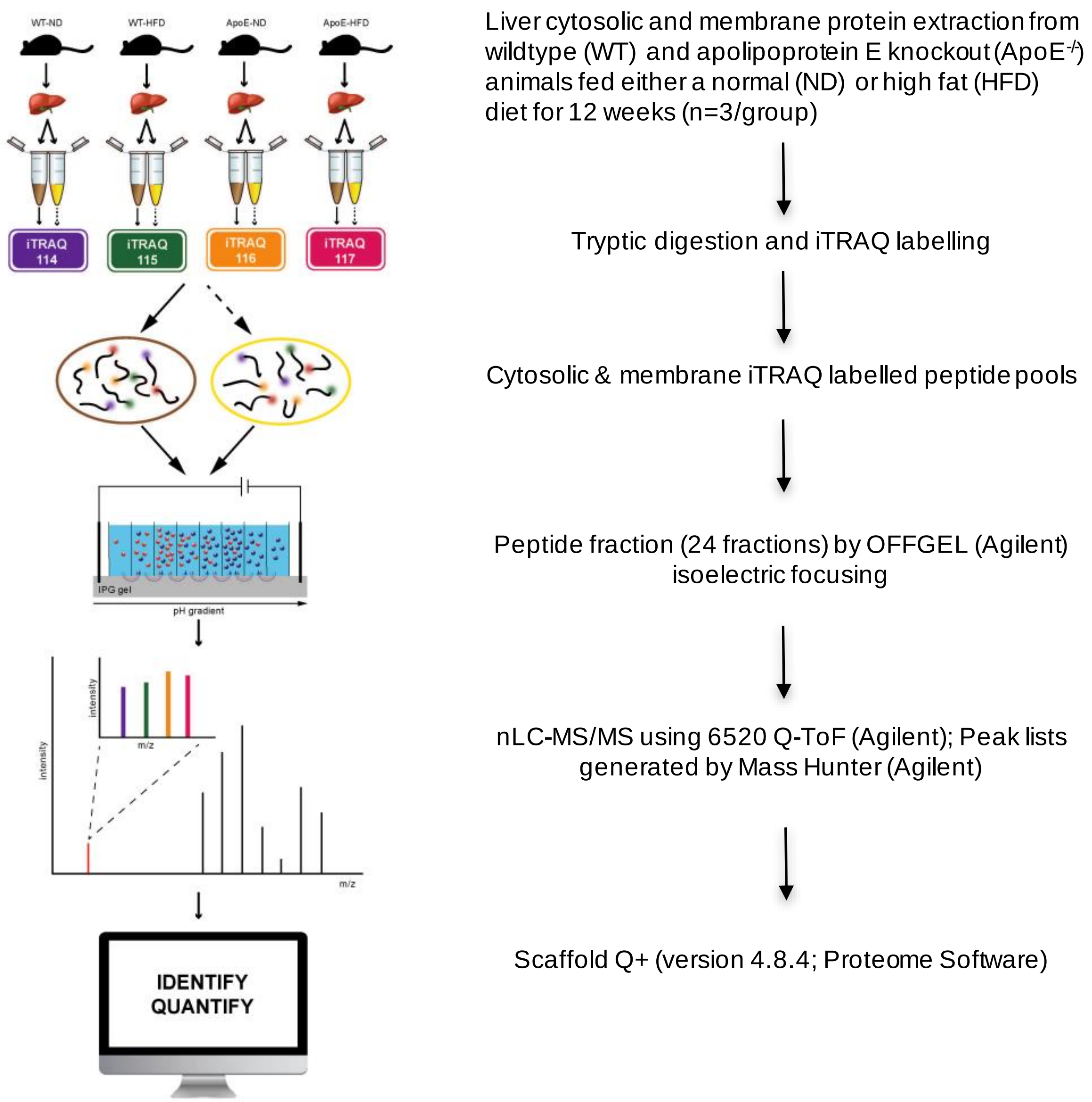

Fig. S1. Proteomic workflow.

Supplementary Figure 2

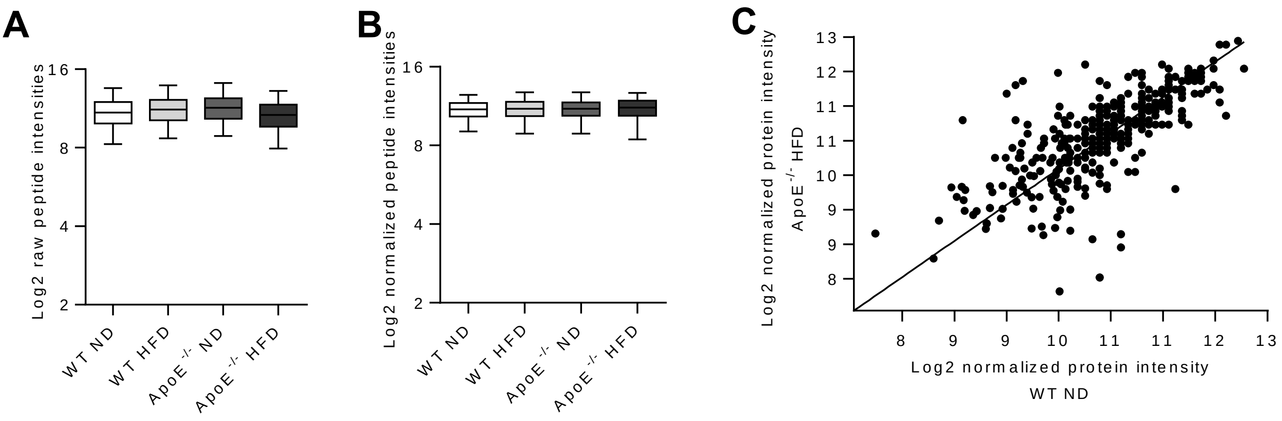

Fig. S2. iTRAQ-based proteomic analysis of murine liver membrane and cytosolic proteins. A Raw and B normalized membrane peptide intensities; data are median of 8522 peptides, whiskers illustrate 5th-95th percentiles; datasets were initially normalized using a peptide mean intensity based normalization. C Scatter plot of 349 normalized membrane protein intensities for WT ND vs. ApoE-/- HFD.

Supplementary Figure 3

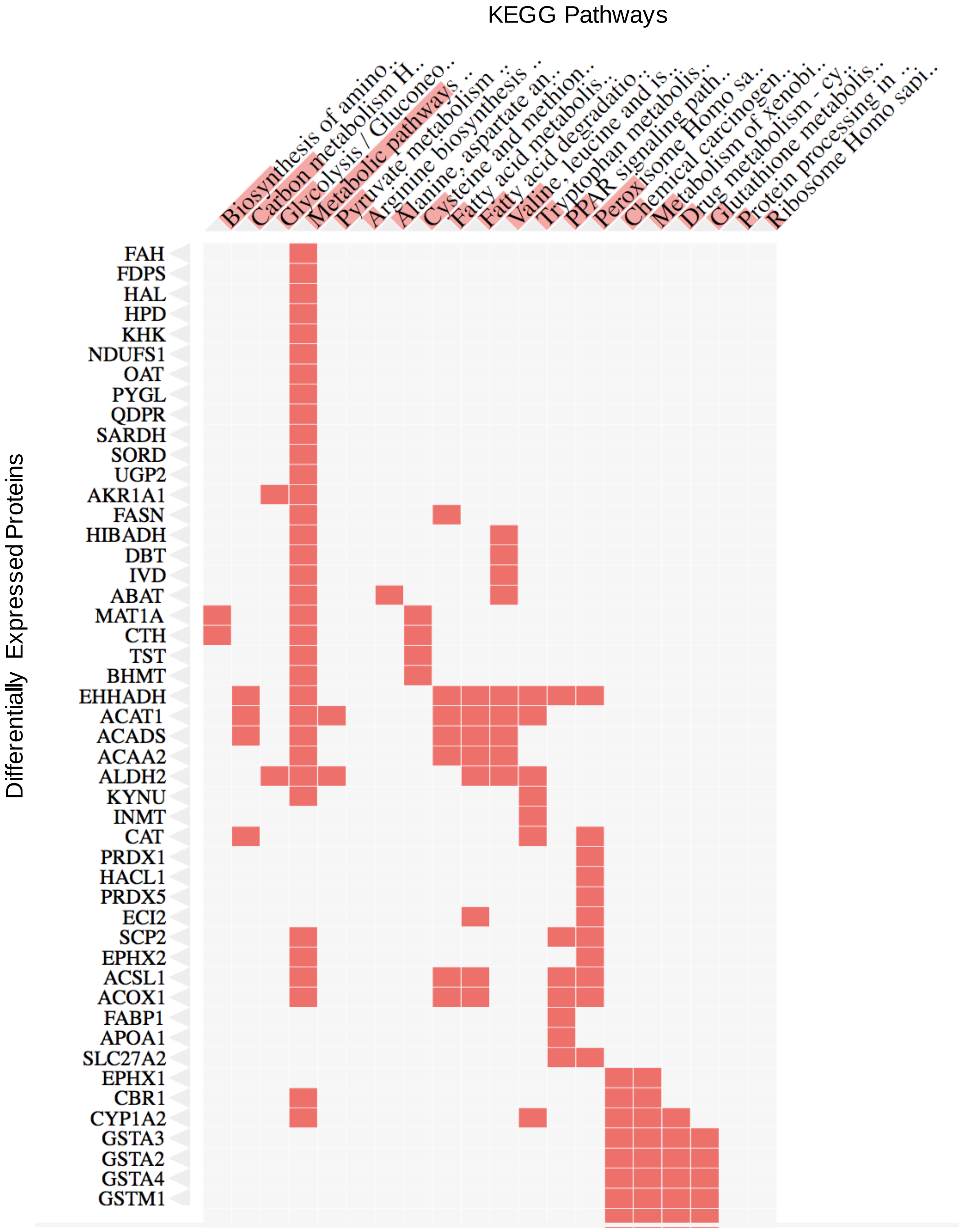

Fig. S3. Clustergram of gene names for differentially expressed proteins significantly overrepresented in KEGG pathways. Data shown for top 20 most significant pathway associations and are clustered according to combined sore for both input proteins and pathways. The Enrichr combined score combines a Fisher's exact P-score and a z-score assessing the deviation from the expected rank (Kuleshov, 2016) to evaluate overrepresentation.

Supplementary Figure 4

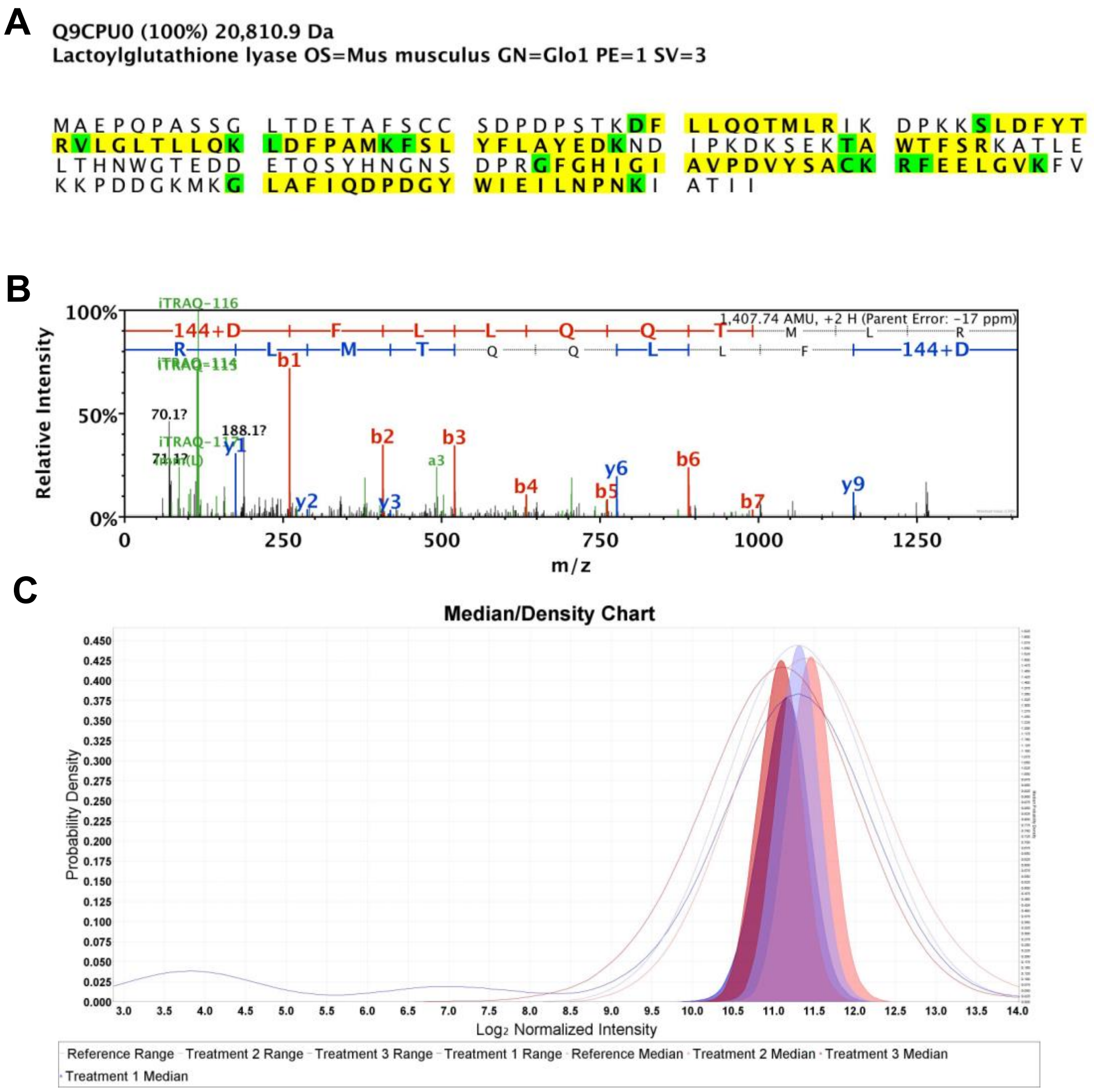

Fig. S4. Proteomic identification of lactoylglutathione lyase (glyoxalase 1; GLO1). **A** MS/MS peptides identified and protein sequence coverage of GLO1. **B** MS/MS spectrum for peptide DFLLQQTMLR. **C** Plot of density distribution estimated using a kernel density function from all spectra. Light blue: WT ND, dark blue: WT HFD, light red: ApoE-/- ND, dark red: ApoE-/- HFD.

Supplementary Figure 5

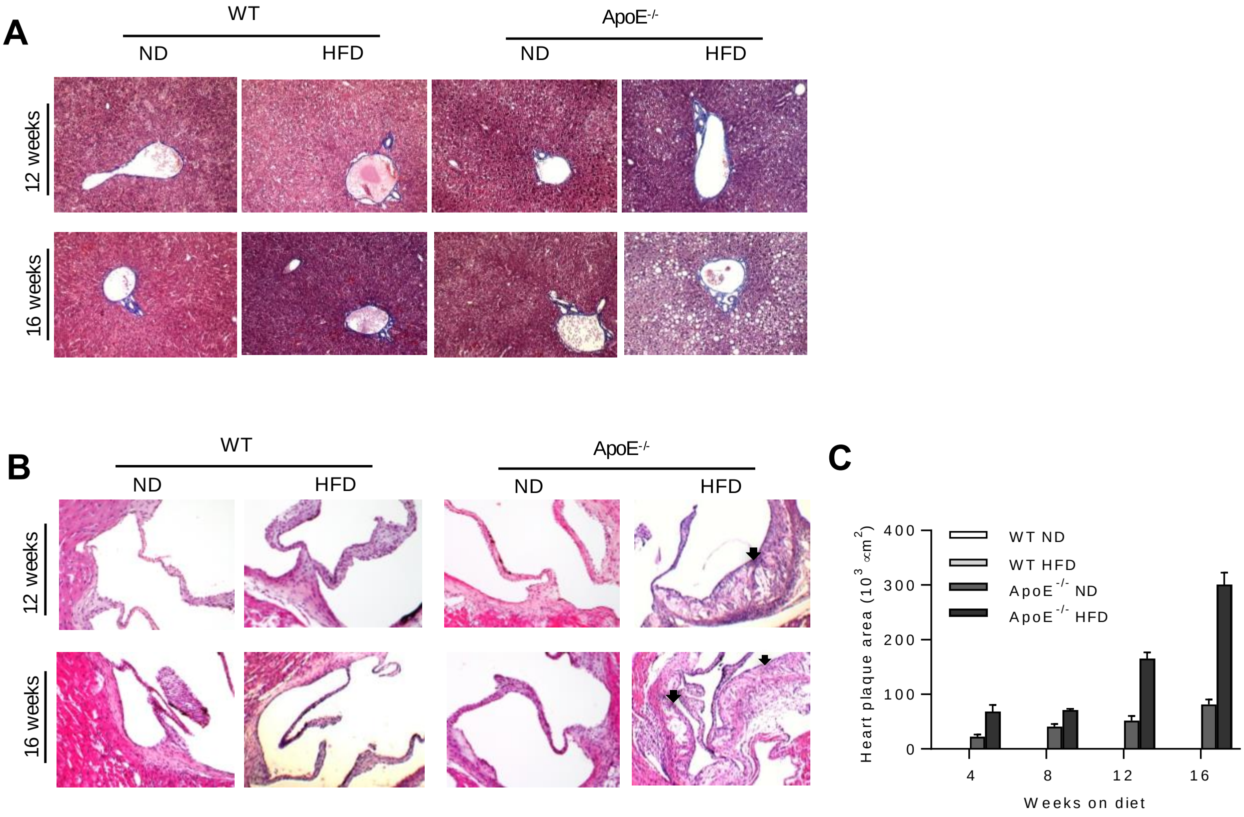

Fig. S5. Hepatic fibrosis and heart pathology after 12 or 16 weeks. **A** Liver sections stained with Masson's trichrome stain from mice. No significant fibrosis is observed after 12 or 16 weeks in any group at any time point. **B** Heart aortic sinus sections taken from mice at 12 or 16 weeks show plaques (arrows) at 12 and 16 weeks. Sections were stained with hematoxylin-eosin; original magnification: 200x. **C** Plaque area in heart sections were quantified using ImageJ software.

Supplementary Figure 6

A

Lactoylglutathione lyase OS=Homo sapiens GN=GLO1 PE=1 SV=4

Database: SwissProt
Score: 200
Nominal mass (M_r): 20764
Calculated pI: 5.12
Taxonomy: Homo sapiens

Sequence similarity is available as an NCBI BLAST search of LGUL_HUMAN against nr.

Search parameters

MS data file: CE_BM_Band_3_BE3_01_1029.mgf
Enzyme: Trypsin: cuts C-term side of KR unless next residue is P.
Variable modifications: Acetyl (K), Acetyl (N-term), Carbamidomethyl (C), Oxidation (M)

Protein sequence coverage: 28%

Matched peptides shown in ***bold red***.

```
  1 MAEPQPPSGG LTDEAALSCC SDADPSTKDF LLQQTMLRVK DPKKSLDFYT
 51 RVLGMTLIQK CDFPIMKFSL YFLAYEDKND IPKEKDEKIA WALSRKATLE
101 LTHNWGTEDD ETQSYHNGNS DPRGFGHIGI AVPDVYSACK RFEELGVKFV
151 KKPDDGKMKG LAFIQDPDGY WIEILNPNKM ATLM
```

Unformatted sequence string: 184 residues (for pasting into other applications).

Sort peptides by ⦿ Residue Number ○ Increasing Mass ○ Decreasing Mass

Show predicted peptides also

Query	Start – End	Observed	Mr(expt)	Mr(calc)	Delta	M	Score	Expect	Rank	U	Peptide
280	29 – 38	632.8800	1263.7454	1263.6645	0.0809	0	62	0.00038	1	U	K.DFLLQQTMLR.V
281	29 – 38	632.8970	1263.7794	1263.6645	0.1149	0	59	0.00081	1	U	K.DFLLQQTMLR.V
286	29 – 38	640.8350	1279.6554	1279.6595	-0.0040	0	68	8.9e-05	1	U	K.DFLLQQTMLR.V + Oxidation (M)
287	29 – 38	640.8630	1279.7114	1279.6595	0.0520	0	60	0.00063	1	U	K.DFLLQQTMLR.V + Oxidation (M)
288	29 – 38	640.8680	1279.7214	1279.6595	0.0620	0	75	2.1e-05	1	U	K.DFLLQQTMLR.V + Oxidation (M)
149	45 – 51	451.2560	900.4974	900.4341	0.0633	0	28	1.2	1	U	K.SLDFYTR.V
188	52 – 60	501.8110	1001.6074	1001.5943	0.0131	0	41	0.06	1	U	R.VLGMTLIQK.C
352	68 – 78	698.5460	1395.0774	1394.6758	0.4017	0	17	14	1	U	K.FSLYFLAYEDK.N
85	89 – 95	408.7360	815.4574	815.4653	-0.0079	0	41	0.073	1	U	K.IAWALSR.K
186	141 – 148	489.2580	976.5014	976.5342	-0.0327	1	46	0.017	1	U	K.RFEELGVK.F
86	142 – 148	411.2300	820.4454	820.4331	0.0124	0	11	52	7	U	R.FEELGVK.F

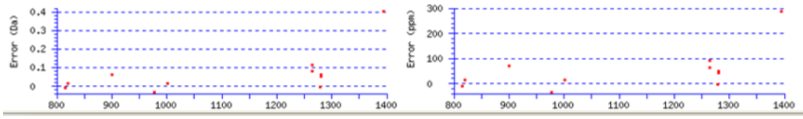

B

MS/MS Fragmentation of **DFLLQQTMLR**
Found in **LGUL_HUMAN** in **SwissProt**, Lactoylglutathione lyase OS=Homo sapiens GN=GLO1 PE=1 SV=4

Match to Query 280: 1263.745448 from(632.880000,2+) intensity(1065786.0000) index(497)
Title: Cmpd 498, +MSn(632.88), 34.0 min
Data file CE_BM_Band_3_BE3_01_1029.mgf

Click mouse within plot area to zoom in by factor of two about that point
Or, Plot from 150 to 1100 Da Full range
Label all possible matches ○ Label matches used for scoring ⦿
Show Y-axis ☐

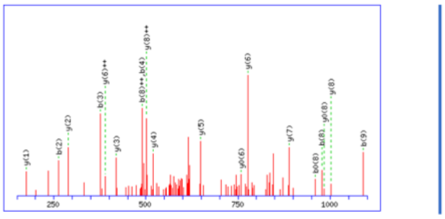


```
Monoisotopic mass of neutral peptide Mr(calc): 1263.6645
Ions Score: 62  Expect: 0.00038
Matches : 19/94 fragment ions using 27 most intense peaks   (help)
```

#	b	b^{++}	b^{*}	b^{*++}	b^{0}	b^{0++}	Seq.	y	y^{++}	y^{*}	y^{*++}	y^{0}	y^{0++}	#
1	116.0342	58.5207			98.0237	49.5155	D							10
2	263.1026	132.0550			245.0921	123.0497	F	1149.6449	575.3261	1132.6183	566.8128	1131.6343	566.3208	9
3	376.1867	188.5970			358.1761	179.5917	L	1002.5765	501.7919	985.5499	493.2786	984.5659	492.7866	8
4	489.2708	245.1390			471.2602	236.1337	L	889.4924	445.2498	872.4659	436.7366	871.4818	436.2446	7
5	617.3293	309.1683	600.3028	300.6550	599.3188	300.1630	Q	776.4083	388.7078	759.3818	380.1945	758.3978	379.7025	6
6	745.3879	373.1976	728.3614	364.6843	727.3774	364.1923	Q	648.3498	324.6785	631.3232	316.1652	630.3392	315.6732	5
7	846.4356	423.7214	829.4090	415.2082	828.4250	414.7162	T	520.2912	260.6492	503.2646	252.1360	502.2806	251.6439	4
8	977.4761	489.2417	960.4495	480.7284	959.4655	480.2364	M	419.2435	210.1254	402.2170	201.6121			3
9	1090.5601	545.7837	1073.5336	537.2704	1072.5496	536.7784	L	288.2030	144.6051	271.1765	136.0919			2
10							R	175.1190	88.0631	158.0924	79.5498			1

Fig. S6. Mass spectrometry confirms immunoprecipitated band as GLO1. **A** MASCOT identification of GLO1 with 28% sequence coverage and 6 peptide sequences. **B** MS/MS spectrum for peptide DFLLQQTMLR.

Supplementary Figure 7

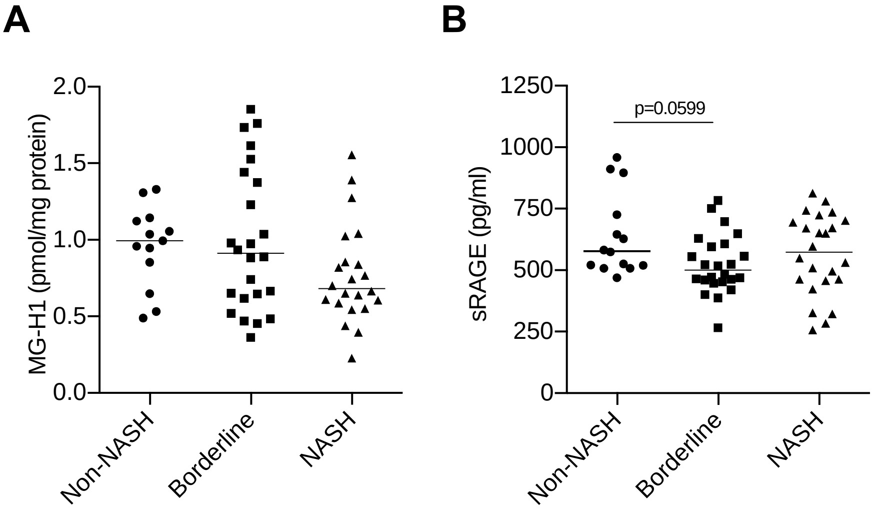

Fig. S7. Serum levels of MG-H1 and sRAGE in adult NAFLD patients. **A** Serum levels of the major methylglyoxal-derived advance glycated endproduct, hydroimidazolone MG-H1, in (n=59) adult NAFLD patients with median illustrated. **B** Serum levels of the soluble receptor for advanced glycation endproducts (sRAGE). Measured in (n=62) adult NAFLD patients with median illustrated.

Supplementary Table 1

Table S1. Differentially expressed proteins in liver cytosolic fraction

Accession	Protein Name	MW	Wt-ND	Wt-HFD	ApoE-ND	ApoE-HFD	Kruskal-Wallis	Permutation
P06801	NADP-dependent malic enzyme	64 kDa	-1.09	-1.00	-0.11	-2.72	< 0.0001	0.00023
Q64471	Glutathione S-transferase theta-1	27 kDa	0.00	-1.91	0.07	-2.15	0.009	0.00089
**P30115**	**Glutathione S-transferase A3**	**25 kDa**	**0.00**	**-1.29**	**0.46**	**-1.49**	**< 0.0001**	**< 0.0001**
Q8VCN5	Cystathionine gamma-lyase	44 kDa	-0.05	-1.42	-0.23	-1.47	0.0012	0.00016
Q9DBE0	Cysteine sulfinic acid decarboxylase	55 kDa	0.04	-1.19	0.38	-1.31	< 0.0001	< 0.0001
Q8BVI4	Dihydropteridine reductase	26 kDa	0.10	-0.74	-0.97	-1.24	0.0044	0.005
P10648	Glutathione S-transferase A2	26 kDa	0.09	-1.15	0.21	-1.23	< 0.0001	< 0.0001
**P19096**	**Fatty acid synthase**	**272 kDa**	**-0.13**	**-0.02**	**-0.68**	**-1.15**	**< 0.0001**	**< 0.0001**
**P10649**	**Glutathione S-transferase Mu 1**	**26 kDa**	**-0.02**	**-0.95**	**0.04**	**-1.11**	**< 0.0001**	**< 0.0001**
P13707	Glycerol-3-phosphate dehydrogenase [NAD+],	38 kDa	-0.06	0.09	0.27	-1.01	< 0.0001	< 0.0001
P40936	Indolethylamine N-methyltransferase	29 kDa	0.00	0.30	0.37	-0.98	0.0024	< 0.0001
P24472	Glutathione S-transferase A4	26 kDa	0.00	-0.38	0.00	-0.98	< 0.0001	< 0.0001
P53657	Pyruvate kinase isozymes R/L	62 kDa	0.05	0.31	-0.58	-0.94	0.013	0.002
**P12710**	**Fatty acid-binding protein, liver**	**14 kDa**	**-0.01**	**-0.44**	**-0.01**	**-0.90**	**< 0.0001**	**< 0.0001**
**P00329**	**Alcohol dehydrogenase 1**	**40 kDa**	**-0.02**	**-0.18**	**0.27**	**-0.90**	**< 0.0001**	**< 0.0001**
**P16015**	**Carbonic anhydrase 3**	**29 kDa**	**-0.02**	**0.39**	**0.31**	**-0.87**	**< 0.0001**	**< 0.0001**
**P35505**	**Fumarylacetoacetase**	**46 kDa**	**0.05**	**-0.94**	**-0.28**	**-0.87**	**0.00023**	**0.0028**
P24549	Retinal dehydrogenase 1	54 kDa	0.00	-0.23	0.51	-0.82	< 0.0001	< 0.0001
Q920E5	Farnesyl pyrophosphate synthase	41 kDa	-0.06	-1.38	0.01	-0.71	0.0011	< 0.0001
**Q64442**	**Sorbitol dehydrogenase**	**38 kDa**	**-0.02**	**-0.08**	**0.11**	**-0.71**	**< 0.0001**	**< 0.0001**
P34884	Macrophage migration inhibitory factor	12 kDa	0.03	-0.64	0.19	-0.68	0.013	0.015
O35945	Aldehyde dehydrogenase, cytosolic 1	55 kDa	0.01	-0.23	0.45	-0.66	0.018	0.0022
Q91ZJ5	UTP--glucose-1-phosphate uridylyltransferase	57 kDa	0.04	-0.51	-0.12	-0.66	0.02	0.015
P35700	Peroxiredoxin-1	22 kDa	0.00	-0.97	-0.30	-0.64	0.00016	0.00048
**P17182**	**Alpha-enolase**	**47 kDa**	**-0.02**	**-0.13**	**0.04**	**-0.59**	**< 0.0001**	**< 0.0001**

Q8BH00	Aldehyde dehydrogenase family 8 member A1	54 kDa	0.05	-0.06	-0.11	-0.57	0.0014	< 0.0001
**Q91Y97**	**Fructose-bisphosphate aldolase B**	**39 kDa**	**-0.03**	**-0.15**	**0.11**	**-0.57**	**< 0.0001**	**< 0.0001**
**P52760**	**Ribonuclease UK114**	**14 kDa**	**0.02**	**-0.25**	**0.12**	**-0.53**	**< 0.0001**	**0.005**
P50247	Adenosylhomocysteinase	48 kDa	-0.01	0.06	0.03	-0.43	< 0.0001	0.00039
**P16858**	**Glyceraldehyde-3-phosphate dehydrogenase**	**36 kDa**	**-0.01**	**0.04**	**-0.14**	**-0.41**	**0.00016**	**0.018**
**Q9ET01**	**Glycogen phosphorylase, liver form.**	**97 kDa**	**-0.02**	**0.09**	**-0.37**	**-0.40**	**0.00024**	**0.0058**
P35492	Histidine ammonia-lyase	72 kDa	0.04	-0.91	-0.52	-0.36	< 0.0001	0.00038
O88844	Isocitrate dehydrogenase [NADP] cytoplasmic	47 kDa	-0.02	-0.22	0.03	-0.26	0.0018	0.0016
**P24270**	**Catalase.**	**60 kDa**	**-0.12**	**-1.01**	**0.02**	**-0.25**	**0.0047**	**0.00088**
Q9CPU0	Lactoylglutathione lyase	21 kDa	0.00	-0.22	0.17	-0.25	0.013	0.014
Q9QXF8	Glycine N-methyltransferase.	33 kDa	-0.02	-0.47	0.01	-0.19	0.00083	0.00034
P62984	Ubiquitin-60S ribosomal protein L40	15 kDa	0.00	-0.43	0.20	-0.18	0.02	0.012
P62983	Ubiquitin-40S ribosomal protein S27a	18 kDa	0.00	-0.43	0.20	-0.18	0.02	0.012
**Q63836**	**Selenium-binding protein 2**	**53 kDa**	**-0.03**	**0.21**	**0.43**	**-0.17**	**< 0.0001**	**< 0.0001**
**Q00623**	**Apolipoprotein A-I**	**31 kDa**	**-0.01**	**0.37**	**-0.75**	**-0.12**	**< 0.0001**	**< 0.0001**
**P11588**	**Major urinary protein 1**	**21 kDa**	**-0.01**	**0.32**	**1.03**	**-0.09**	**< 0.0001**	**< 0.0001**
Q9JII6	Alcohol dehydrogenase [NADP+]	37 kDa	-0.01	-0.11	0.28	-0.06	0.0033	0.011
P09411	Phosphoglycerate kinase 1.	45 kDa	0.01	-0.29	-0.20	-0.01	0.0088	0.018
P17742	Peptidyl-prolyl cis-trans isomerase A	18 kDa	0.01	-0.59	0.04	-0.01	0.00071	0.001
P48758	Carbonyl reductase [NADPH] 1	31 kDa	0.02	-0.64	-0.39	0.02	0.0017	0.00073
Q8C196	Carbamoyl-phosphate synthase [ammonia],	165 kDa	-0.01	0.16	-0.43	0.04	< 0.0001	< 0.0001
Q923D2	Flavin reductase (NADPH)	22 kDa	0.01	-0.27	0.45	0.16	< 0.0001	0.001
P17751	Triosephosphate isomerase	32 kDa	-0.01	-0.26	-0.14	0.17	< 0.0001	0.00067
Q8VC30	Bifunctional ATP-dependent dihydroxyacetone	60 kDa	-0.03	0.78	0.05	0.19	< 0.0001	< 0.0001
P38647	Stress-70 protein, mitochondrial	74 kDa	0.04	-0.18	-0.38	0.27	0.00025	0.00015
P10639	Thioredoxin	12 kDa	-0.11	0.01	0.44	0.27	0.0039	0.004
P99029	Peroxiredoxin-5, mitochondrial	22 kDa	-0.02	-0.16	0.01	0.29	0.0023	0.0022
Q9CXF0	Kynureninase	52 kDa	-0.01	0.25	-0.66	0.34	0.0024	< 0.0001
P61922	4-aminobutyrate aminotransferase, mitochondrial	56 kDa	0.02	-0.10	-0.35	0.38	0.001	0.0001
P63038	60 kDa heat shock protein, mitochondrial	61 kDa	-0.03	-0.01	-0.18	0.40	< 0.0001	< 0.0001

**Q07417**	**Short-chain specific acyl-CoA dehydrogenase,**	**45 kDa**	**0.00**	**-0.44**	**-0.26**	**0.40**	**< 0.0001**	**< 0.0001**
Q9DCW4	Electron transfer flavoprotein subunit beta	28 kDa	-0.01	-0.20	-0.26	0.40	< 0.0001	< 0.0001
**Q99LB7**	**Sarcosine dehydrogenase, mitochondrial**	**102 kDa**	**0.02**	**0.17**	**-0.36**	**0.42**	**< 0.0001**	**< 0.0001**
Q9D819	Inorganic pyrophosphatase	33 kDa	-0.01	-0.01	-0.07	0.42	0.0068	0.0042
P97328	Ketohexokinase	33 kDa	-0.02	0.69	0.44	0.44	0.01	0.0013
P48036	Annexin A5	36 kDa	0.01	-0.22	-0.17	0.47	< 0.0001	0.002
P10126	Elongation factor 1-alpha 1	50 kDa	-0.05	0.24	0.15	0.51	0.0044	0.019
Q8QZT1	Acetyl-CoA acetyltransferase, mitochondrial	45 kDa	0.00	-0.04	-0.27	0.52	< 0.0001	0.001
P53395	Lipoamide acyltransferase component of branched-chain	53 kDa	0.01	0.51	-0.86	0.54	0.018	0.00091
Q9JHI5	Isovaleryl-CoA dehydrogenase, mitochondrial	46 kDa	-0.03	-0.22	-0.08	0.56	0.0018	0.00016
**P08249**	**Malate dehydrogenase, mitochondrial**	**36 kDa**	**0.01**	**-0.06**	**-0.17**	**0.58**	**< 0.0001**	**< 0.0001**
Q9D0S9	Histidine triad nucleotide-binding protein 2,	17 kDa	-0.03	0.37	-0.07	0.58	0.0093	0.016
Q99LC5	Electron transfer flavoprotein subunit alpha,	35 kDa	0.02	-0.11	-0.02	0.61	< 0.0001	0.019
P40124	Adenylyl cyclase-associated protein 1	52 kDa	-0.03	0.10	-0.50	0.68	0.014	0.0032
P05201	Aspartate aminotransferase, cytoplasmic	46 kDa	0.01	-0.52	-0.17	0.69	< 0.0001	< 0.0001
Q05920	Pyruvate carboxylase, mitochondrial	130 kDa	-0.02	0.32	0.04	0.69	< 0.0001	< 0.0001
P47738	Aldehyde dehydrogenase, mitochondrial	57 kDa	-0.01	0.12	0.08	0.70	< 0.0001	< 0.0001
P63028	Translationally-controlled tumor protein	19 kDa	-0.04	0.55	-1.22	0.76	0.0074	0.014
Q91X83	S-adenosylmethionine synthase isoform type-1	43 kDa	0.00	0.28	-0.17	0.77	< 0.0001	< 0.0001
**P60710**	**Actin, cytoplasmic 1**	**42 kDa**	**-0.02**	**0.09**	**0.26**	**0.77**	**< 0.0001**	**0.0009**
O08997	Copper transport protein ATOX1	7 kDa	0.01	0.46	0.09	0.84	0.0032	0.00032
**P09103**	**Protein disulfide-isomerase**	**57 kDa**	**-0.03**	**0.27**	**0.14**	**0.85**	**< 0.0001**	**0.00083**
**Q8BWT1**	**3-ketoacyl-CoA thiolase, mitochondrial**	**42 kDa**	**-0.01**	**0.08**	**-0.07**	**0.85**	**< 0.0001**	**< 0.0001**
O35423	Serine--pyruvate aminotransferase, mitochondrial	46 kDa	0.03	-1.39	-0.91	0.90	0.016	0.00023
P22599	Alpha-1-antitrypsin 1-2	46 kDa	0.02	0.03	0.63	0.93	0.0042	0.002
Q9Z2V4	Phosphoenolpyruvate carboxykinase, cytosolic [GTP]	69 kDa	0.02	0.02	-0.04	1.00	0.00033	< 0.0001
**P14211**	**Calreticulin**	**48 kDa**	**-0.35**	**-0.20**	**0.43**	**1.05**	**0.00089**	**0.00043**
P00920	Carbonic anhydrase 2	29 kDa	-0.04	0.27	0.97	1.11	< 0.0001	0.0014

**P20029**	**78 kDa glucose-regulated protein**	**72 kDa**	**-0.03**	**-0.10**	**-0.14**	**1.16**	**< 0.0001**	**< 0.0001**
P56480	ATP synthase subunit beta, mitochondrial	56 kDa	-0.01	-0.58	-0.46	1.17	< 0.0001	< 0.0001
Q61838	Alpha-2-macroglobulin	166 kDa	0.12	-0.09	0.78	1.33	0.00061	0.01
**Q921I1**	**Serotransferrin**	**77 kDa**	**0.00**	**0.15**	**-0.15**	**1.35**	**< 0.0001**	**< 0.0001**
**P08003**	**Protein disulfide-isomerase A4**	**72 kDa**	**0.01**	**0.37**	**0.06**	**1.38**	**0.0035**	**< 0.0001**
**P29758**	**Ornithine aminotransferase, mitochondrial**	**48 kDa**	**-0.01**	**0.15**	**0.36**	**1.49**	**< 0.0001**	**< 0.0001**
P26443	Glutamate dehydrogenase 1, mitochondrial	61 kDa	-0.02	0.56	-0.25	1.56	< 0.0001	< 0.0001
Q91X72	Hemopexin	51 kDa	-0.01	-0.29	-2.19	1.70	0.011	0.00079

All proteins were identified and relatively quantified by 2 or more unique peptides in all three mass spectrometry experiments; and were significantly different by both randomized permutation and Kruskal-Wallis test with Benjamini-Hochberg FDR used for multiple testing corrections. Proteins identified in both cytosol and membrane fractions are in bold. Data are expressed as log2 fold change and are sorted by changes in the ApoE-HFD.

Supplementary Table 2

Table S2. Differentially expressed proteins in liver membrane fraction

Accession	Protein Name	MW	Wt-ND	Wt-HFD	ApoE-ND	ApoE-HFD	Kruskal-Wallis	Permutation
Q8VCW8	Acyl-CoA synthetase family member 2, mitochondrial	68 kDa	-0.04	-2.06	-1.22	-2.49	0.0097	0.009
Q64459	Cytochrome P450 3A11	58 kDa	0.00	-1.33	0.39	-2.33	< 0.0001	< 0.0001
P08226	Apolipoprotein E	36 kDa	-0.08	0.94	-1.15	-1.89	0.0051	0.0034
Q9QXE0	2-hydroxyacyl-CoA lyase 1	64 kDa	0.10	-0.28	-0.10	-1.77	0.0074	< 0.0001
**P12710**	**Fatty acid-binding protein, liver**	**14 kDa**	**-0.01**	**-0.42**	**0.45**	**-1.64**	**< 0.0001**	**< 0.0001**
Q8VCH0	3-ketoacyl-CoA thiolase B, peroxisomal	44 kDa	-0.09	0.20	0.05	-1.47	0.0031	< 0.0001
Q91WL5	Cytochrome P450 4A12A	58 kDa	0.06	-0.36	0.05	-1.45	0.002	0.0024
Q64458	Cytochrome P450 2C29	56 kDa	0.00	-0.28	0.41	-1.31	< 0.0001	< 0.0001
**P16015**	**Carbonic anhydrase 3**	**29 kDa**	**-0.02**	**0.20**	**0.58**	**-1.30**	**< 0.0001**	**< 0.0001**
Q9D379	Epoxide hydrolase 1	53 kDa	-0.02	-0.50	-0.20	-1.03	< 0.0001	0.0041
**Q9ET01**	**Glycogen phosphorylase, liver form.**	**97 kDa**	**0.03**	**0.16**	**-0.05**	**-0.99**	**< 0.0001**	**< 0.0001**
**P00329**	**Alcohol dehydrogenase 1**	**40 kDa**	**0.01**	**-1.12**	**-0.74**	**-0.97**	**< 0.0001**	**< 0.0001**
P41216	Long-chain-fatty-acid--CoA ligase 1	78 kDa	-0.02	-0.07	-0.01	-0.97	< 0.0001	< 0.0001
P19157	Glutathione S-transferase P 1	24 kDa	-0.08	0.06	-0.03	-0.86	0.0084	0.00012
Q8VCT4	Carboxylesterase 3	62 kDa	-0.02	-0.10	-0.18	-0.80	< 0.0001	< 0.0001
**P24270**	**Catalase.**	**60 kDa**	**0.00**	**-0.12**	**0.17**	**-0.77**	**< 0.0001**	**< 0.0001**
**P30115**	**Glutathione S-transferase A3**	**25 kDa**	**0.01**	**-0.63**	**0.17**	**-0.76**	**0.00057**	**0.001**
P00186	Cytochrome P450 1A2	58 kDa	-0.02	-0.52	0.46	-0.76	< 0.0001	< 0.0001
P17717	UDP-glucuronosyltransferase 2B17	61 kDa	0.00	-0.55	-0.16	-0.74	< 0.0001	< 0.0001
**Q99LB7**	**Sarcosine dehydrogenase, mitochondrial**	**102 kDa**	**-0.04**	**0.26**	**0.42**	**-0.71**	**< 0.0001**	**< 0.0001**
P34914	Epoxide hydrolase 2	62 kDa	0.01	0.38	0.38	-0.69	< 0.0001	< 0.0001
P25688	Uricase	35 kDa	0.01	-0.26	-0.53	-0.68	< 0.0001	0.002

O88451	Retinol dehydrogenase 7	36 kDa	-0.01	0.04	-0.34	-0.68	0.0017	0.00065
**Q63836**	**Selenium-binding protein 2**	**53 kDa**	**-0.07**	**0.43**	**0.52**	**-0.67**	**0.00019**	**0.00015**
P17563	Selenium-binding protein 1	52 kDa	-0.07	0.43	0.52	-0.67	0.00019	0.00015
Q9R0H0	Peroxisomal acyl-coenzyme A oxidase 1	75 kDa	0.02	-0.41	0.09	-0.62	< 0.0001	< 0.0001
**P29758**	**Ornithine aminotransferase, mitochondrial**	**48 kDa**	**-0.23**	**-0.11**	**0.52**	**-0.61**	**< 0.0001**	**0.011**
**Q07417**	**Short-chain specific acyl-CoA dehydrogenase,**	**45 kDa**	**0.03**	**0.12**	**0.81**	**-0.53**	**0.0014**	**0.00089**
P32020	Non-specific lipid-transfer protein	59 kDa	-0.02	-0.02	0.48	-0.52	< 0.0001	< 0.0001
Q99PG0	Arylacetamide deacetylase.	45 kDa	-0.02	0.29	-0.09	-0.52	0.00037	0.003
Q9DBM2	Peroxisomal bifunctional enzyme	78 kDa	-0.02	0.28	0.27	-0.51	< 0.0001	< 0.0001
P33267	Cytochrome P450 2F2	56 kDa	0.00	-0.39	-0.14	-0.49	0.00014	< 0.0001
P52760	Ribonuclease UK114	14 kDa	0.02	0.17	0.16	-0.46	0.0027	< 0.0001
Q03265	ATP synthase subunit alpha, mitochondrial	60 kDa	0.00	-0.01	-0.13	-0.41	0.0035	0.003
**P10649**	**Glutathione S-transferase Mu 1**	**26 kDa**	**-0.01**	**-0.39**	**0.40**	**-0.37**	**< 0.0001**	**0.0001**
**P11588**	**Major urinary protein 1**	**21 kDa**	**0.01**	**0.01**	**0.62**	**-0.37**	**< 0.0001**	**0.00031**
**Q8BWT1**	**3-ketoacyl-CoA thiolase, mitochondrial**	**42 kDa**	**-0.01**	**0.21**	**0.26**	**-0.29**	**< 0.0001**	**0.011**
P58710	L-gulonolactone oxidase	50 kDa	0.01	-0.32	0.37	-0.18	0.00073	0.00055
Q91YI0	Argininosuccinate lyase	52 kDa	0.00	0.16	-0.47	-0.08	0.0029	0.00035
**P17182**	**Alpha-enolase**	**47 kDa**	**-0.01**	**0.22**	**-0.40**	**-0.02**	**< 0.0001**	**< 0.0001**
**P08249**	**Malate dehydrogenase, mitochondrial**	**36 kDa**	**-0.02**	**-0.17**	**0.56**	**-0.02**	**0.0032**	**0.004**
Q91VD9	NADH-ubiquinone oxidoreductase 75 kDa subunit,	80 kDa	0.03	-0.74	-0.43	0.06	0.011	0.0014
P08113	Endoplasmin	92 kDa	0.00	-0.39	0.00	0.14	< 0.0001	0.0018
P60710	Actin, cytoplasmic 1	42 kDa	0.00	-0.31	-0.20	0.22	0.00042	< 0.0001
P62855	40S ribosomal protein S26.	13 kDa	-0.02	-0.66	-0.51	0.25	0.012	0.003
P63323	40S ribosomal protein S12.	15 kDa	0.04	-0.54	-0.43	0.26	0.01	0.002
O35488	Very long-chain acyl-CoA synthetase	70 kDa	-0.01	0.48	-0.11	0.26	0.00057	0.001
O35490	Betaine--homocysteine S-methyltransferase 1.	45 kDa	0.01	0.84	-0.10	0.27	< 0.0001	< 0.0001
P27773	Protein disulfide-isomerase A3	57 kDa	-0.02	-0.25	-0.02	0.28	< 0.0001	0.005
**P14211**	**Calreticulin**	**48 kDa**	**0.02**	**-0.40**	**-0.26**	**0.28**	**< 0.0001**	**0.00074**
P00405	Cytochrome c oxidase subunit 2	26 kDa	-0.02	-0.31	-0.05	0.29	0.0043	0.0022

**P09103**	**Protein disulfide-isomerase**	**57 kDa**	**-0.01**	**-0.04**	**-0.21**	**0.36**	**< 0.0001**	**< 0.0001**
Q91YQ5	Dolichyl-diphosphooligosaccharide--protein	69 kDa	-0.04	-0.37	-0.10	0.43	0.011	0.00094
P52196	Thiosulfate sulfurtransferase	33 kDa	-0.04	0.08	-0.44	0.43	0.014	0.011
P62918	60S ribosomal protein L8.	28 kDa	-0.02	0.30	-0.49	0.46	0.00012	0.0019
P12787	Cytochrome c oxidase subunit 5A, mitochondrial	16 kDa	0.03	0.11	-0.05	0.54	0.00062	< 0.0001
**P20029**	**78 kDa glucose-regulated protein**	**72 kDa**	**-0.01**	**-0.04**	**-0.07**	**0.60**	**< 0.0001**	**< 0.0001**
**P08003**	**Protein disulfide-isomerase A4**	**72 kDa**	**0.01**	**-0.15**	**-0.32**	**0.60**	**< 0.0001**	**< 0.0001**
Q99L13	3-hydroxyisobutyrate dehydrogenase, mitochondrial	35 kDa	-0.15	0.82	0.61	0.62	0.0057	0.003
P05202	Aspartate aminotransferase, mitochondrial	47 kDa	-0.03	0.19	0.04	0.62	< 0.0001	< 0.0001
**Q91Y97**	**Fructose-bisphosphate aldolase B**	**39 kDa**	**0.01**	**0.64**	**-0.27**	**0.64**	**< 0.0001**	**< 0.0001**
P49429	4-hydroxyphenylpyruvate dioxygenase	45 kDa	-0.06	0.28	-0.08	0.65	< 0.0001	< 0.0001
**Q921I1**	**Serotransferrin**	**77 kDa**	**-0.05**	**0.04**	**-0.12**	**0.69**	**< 0.0001**	**0.00038**
**Q64442**	**Sorbitol dehydrogenase**	**38 kDa**	**-0.10**	**0.64**	**0.30**	**0.71**	**0.01**	**0.011**
**P19096**	**Fatty acid synthase**	**272 kDa**	**0.01**	**1.01**	**0.31**	**0.73**	**0.015**	**0.004**
Q8BP67	60S ribosomal protein L24.	18 kDa	0.10	0.09	0.12	0.77	0.0028	0.0017
Q61176	Arginase-1	35 kDa	-0.03	-0.05	-0.04	0.81	< 0.0001	< 0.0001
Q9WUR2	Enoyl-CoA delta isomerase 2, mitochondrial	43 kDa	0.00	0.44	0.14	0.81	0.014	0.0015
Q99PL5	Ribosome-binding protein 1	173 kDa	0.02	0.02	0.13	0.83	< 0.0001	< 0.0001
P12970	60S ribosomal protein L7a	30 kDa	-0.01	0.65	0.27	0.89	0.00065	0.001
**P35505**	**Fumarylacetoacetase**	**46 kDa**	**-0.06**	**0.57**	**0.49**	**0.91**	**< 0.0001**	**0.00098**
Q8CFX1	GDH/6PGL endoplasmic bifunctional protein	89 kDa	0.00	0.39	-0.67	0.97	0.0047	0.0004
P97807	Fumarate hydratase, mitochondrial	54 kDa	0.04	0.00	-0.36	1.12	< 0.0001	< 0.0001
**Q00623**	**Apolipoprotein A-I**	**31 kDa**	**-0.41**	**0.30**	**0.72**	**1.12**	**0.0032**	**0.0023**
Q91VA0	Acyl-coenzyme A synthetase ACSM1, mitochondrial	65 kDa	-0.30	0.44	0.66	1.35	0.00023	0.00018
O88569	Heterogeneous nuclear ribonucleoproteins A2/B1	37 kDa	-0.05	0.31	-0.14	1.47	0.011	0.00096
**P16858**	**Glyceraldehyde-3-phosphate dehydrogenase**	**36 kDa**	**-0.10**	**0.84**	**0.67**	**1.59**	**< 0.0001**	**< 0.0001**
P62806	Histone H4.	11 kDa	0.07	-0.89	-1.37	1.78	0.0053	0.00041
P06151	L-lactate dehydrogenase A chain	36 kDa	-0.08	0.84	0.24	2.00	0.0011	< 0.0001
Q8CGP6	Histone H2A type 1-H.	14 kDa	-0.01	-0.44	-0.73	2.17	0.004	< 0.0001

Q8CGP5	Histone H2A type 1-F.	14 kDa	-0.01	-0.44	-0.73	2.17	0.004	< 0.0001

All proteins were identified and relatively quantified by 2 or more unique peptides in all three mass spectrometry experiments; and were significantly different by both randomized permutation and Kruskal-Wallis test with Benjamini-Hochberg FDR used for multiple testing corrections. Proteins identified in both cytosol and membrane fractions are in bold. Data are expressed as log2 fold change and are sorted by changes in the ApoE-HFD.

Supplementary Table 3

Table S3. Correlation of sRAGE with clinical features of NAFLD

Variables	r	P
sRAGE versus NAS	-0.176	0.1706
sRAGE versus inflammation	-0.2169	0.0904
sRAGE versus ballooning	-0.2007	0.1178
sRAGE versus ALT	-0.2009	0.1175
sRAGE versus BMI	0.018	0.8908

Pearson or Spearman correlation was used to analyse the relationship between variables.
Abbreviations: sRAGE, soluble receptor for advanced glycation end products; NAS, NAFLD activity score; ALT, alanine aminotransferase; BMI, body mass index.

Supplementary Table 4

Table S4. Histopathological scoring for individual biopsies from 9 pediatric patients with NAFLD

Patient	Steatosis	Lobular inflammation	Hepatocellular ballooning	Fibrosis
1	3	0	0	1c
2	3	1	0	1c
3	3	1	1	2
4	1	0	1	1
5	1	1	1	3
6	2	0	2	3
7	1	1	0	1
8	1	2	2	3
9	3	1	0	3
